# An aberrant sugar modification of BACE1 blocks its lysosomal targeting in Alzheimer's disease

**DOI:** 10.15252/emmm.201404438

**Published:** 2015-01-15

**Authors:** Yasuhiko Kizuka, Shinobu Kitazume, Reiko Fujinawa, Takashi Saito, Nobuhisa Iwata, Takaomi C Saido, Miyako Nakano, Yoshiki Yamaguchi, Yasuhiro Hashimoto, Matthias Staufenbiel, Hiroyuki Hatsuta, Shigeo Murayama, Hiroshi Manya, Tamao Endo, Naoyuki Taniguchi

**Affiliations:** 1 Disease Glycomics Team, RIKEN-Max Planck Joint Research Center, Global Research Cluster, RIKENWako, Japan; 2 Laboratory for Proteolytic Neuroscience, RIKEN Brain Science InstituteWako, Japan; 3 Department of Genome-based Drug Discovery, Unit of Molecular Medicinal Sciences, Graduate School of Biomedical Sciences, Nagasaki UniversityNagasaki, Japan; 4 Graduate School of Advanced Sciences of Matter, Hiroshima UniversityHigashihiroshima, Hiroshima, Japan; 5 Structural Glycobiology Team, RIKEN-Max Planck Joint Research Center, Global Research Cluster, RIKENWako, Japan; 6 Department of Biochemistry, Fukushima Medical University School of MedicineFukushima, Japan; 7 Novartis Institutes for Biomedical ResearchBasel, Switzerland; 8 Department of Neuropathology, Research Team for Mechanism of Aging, Tokyo Metropolitan Geriatric Hospital and Institute of GerontologyItabashi-ku, Tokyo, Japan; 9 Molecular Glycobiology, Research Team for Mechanism of Aging, Tokyo Metropolitan Geriatric Hospital and Institute of GerontologyItabashi-ku, Tokyo, Japan

**Keywords:** Alzheimer's disease (AD), amyloid-β (Aβ), BACE1, bisecting GlcNAc, GnT-III (*Mgat3*)

## Abstract

The β-site amyloid precursor protein cleaving enzyme-1 (BACE1), an essential protease for the generation of amyloid-β (Aβ) peptide, is a major drug target for Alzheimer's disease (AD). However, there is a concern that inhibiting BACE1 could also affect several physiological functions. Here, we show that BACE1 is modified with bisecting N-acetylglucosamine (GlcNAc), a sugar modification highly expressed in brain, and demonstrate that AD patients have higher levels of bisecting GlcNAc on BACE1. Analysis of knockout mice lacking the biosynthetic enzyme for bisecting GlcNAc, GnT-III (*Mgat3*), revealed that cleavage of Aβ-precursor protein (APP) by BACE1 is reduced in these mice, resulting in a decrease in Aβ plaques and improved cognitive function. The lack of this modification directs BACE1 to late endosomes/lysosomes where it is less colocalized with APP, leading to accelerated lysosomal degradation. Notably, other BACE1 substrates, CHL1 and contactin-2, are normally cleaved in GnT-III-deficient mice, suggesting that the effect of bisecting GlcNAc on BACE1 is selective to APP. Considering that GnT-III-deficient mice remain healthy, GnT-III may be a novel and promising drug target for AD therapeutics.

## Introduction

Alzheimer's disease (AD) is a devastating dementia, with the number of patients now estimated to be ∽0.5% of the global population (Abbott, 2011; Selkoe, 2012). Deposition of amyloid-β (Aβ) peptide in the brain is considered to represent the initial event in disease development (Karran *et al*, 2011). Aβ is generated by the two-step proteolytic cleavage of amyloid precursor protein (APP), which is catalyzed by the β-site APP cleaving enzyme-1 (BACE1, also designated as β-secretase) (Vassar *et al*, 2014) and γ-secretase (De Strooper & Annaert, 2010). However, when APP is cleaved at the α-site within the Aβ sequence by α-secretase, pathogenic Aβ is not generated. Current trials to develop γ-secretase inhibitors have been unsuccessful due to serious side effects, probably as a result of disturbing the signaling of Notch (De Strooper *et al*, 1998), another substrate for γ-secretase. BACE1 protease also has substrates other than APP (Kuhn *et al*, 2012; Vassar *et al*, 2014), including α2,6-sialyltransferase (Kitazume *et al*, 2001), P-selectin glycoprotein ligand-1 (PSGL-1) (Lichtenthaler *et al*, 2003), APP homolog proteins (APLP1 and APLP2) (Eggert *et al*, 2004; Li & Sudhof, 2004; Pastorino *et al*, 2004), low-density lipoprotein receptor-related protein (LRP) (von Arnim *et al*, 2005), voltage-gated sodium channel (Na_v_1) β subunits (Kim *et al*, 2005; Wong *et al*, 2005), neuregulins 1 and 3 (NRG1, 3) (Hu *et al*, 2006; Willem *et al*, 2006), and neural cell adhesion molecules (L1 and CHL1) (Zhou *et al*, 2012). *Bace1*^−/−^ mice display retinal pathology (Cai *et al*, 2012) and changes in NRG1 signaling, leading to a schizophrenia-like phenotype (Savonenko *et al*, 2008) and impaired formation of muscle spindles (Cheret *et al*, 2013). This indicates that BACE1 also has physiological roles in addition to its involvement in the pathogenesis of AD.

Increasing evidence shows that aberrant glycosylation is a critical factor for the development of various diseases (Dennis *et al*, 2009; Godfrey *et al*, 2011; Ohtsubo *et al*, 2005). Lack of glycosylation causes dysfunction of target glycoproteins, including impaired glycoprotein folding (Hebert *et al*, 2005), poor ligand binding of a receptor glycoprotein (Wang *et al*, 2005), or shortened cell surface retention of a glycoprotein (Dennis *et al*, 2009; Ohtsubo *et al*, 2005). Although the roles of glycans in AD pathology remain unclear, most AD-related molecules, including APP and its secretases (a disintegrin and metalloproteinases (ADAMs) and BACE1), carry glycans, highlighting the possibility that Aβ generation could be regulated by their glycosylation.

**Figure 1:**
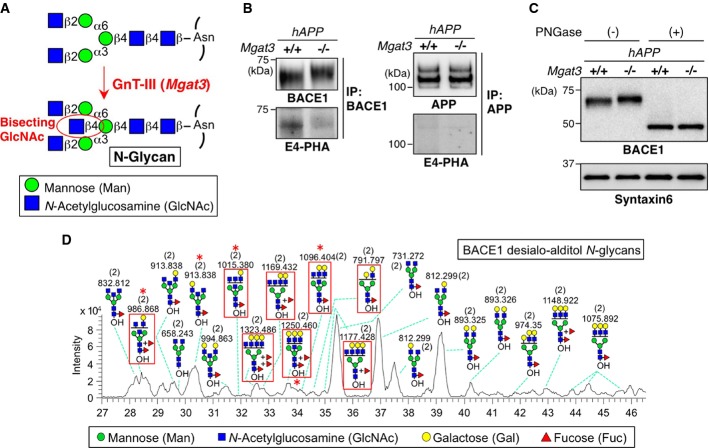
BACE1 is modified with bisecting GlcNAc *in vivo*

Here, we focus on bisecting N-acetylglucosamine (GlcNAc), a unique *N*-glycan structure that is highly expressed in the brain (Fig1A). Although this sugar modification has been suggested to suppress cancer metastasis (Taniguchi *et al*, 2006), its target glycoprotein and the function of bisecting GlcNAc in the brain have not been explored. We have previously found that the glycosyltransferase, GnT-III (encoded by the *MGAT3* gene) (Nishikawa *et al*, 1992), which is the sole biosynthetic enzyme for bisecting GlcNAc modification (Bhattacharyya *et al*, 2002), is upregulated in the brains of AD patients (Akasaka-Manya *et al*, 2010), but how this increase in bisected glycan contributes to AD pathology remained unclear. In this study, we have identified BACE1 as a novel *in vivo* target glycoprotein for this modification. By analyzing the brains of GnT-III (*Mgat3*)-deficient mice, we demonstrate that the sugar modification promotes AD pathogenesis by delaying BACE1 degradation. Considering that *Mgat3*^−/−^ mice show almost no phenotypic abnormality in terms of development, reproduction, hematology, and brain morphology (Orr *et al*, 2013; Priatel *et al*, 1997), our results highlight the possibility of a novel strategy for developing glycosyltransferase-targeted AD therapeutics.

Bisecting GlcNAc modification by GnT-III.

BACE1 or APP was immunoprecipitated from mouse brains and blotted with E4-PHA lectin (lower) or anti-BACE1 or APP antibodies (upper). *hAPP* indicates the APP23 transgenic mouse model for AD.

Proteins from mouse brain membrane fractions were treated with or without PNGase F and then immunoblotted for BACE1 or for syntaxin 6 (loading control).

LC–MS base peak chromatogram of desialo-alditol *N*-glycans derived from mouse brain BACE1. To simplify the results, *N*-glycans were chemically desialylated before LC–MS analysis. BACE1-specific glycans, judged by comparison with *N*-glycan structures from anti-BACE1 IgG (shown in Supplementary Fig S2C), are highlighted by red squares. Asterisks indicate glycans demonstrated by MS/MS analysis to contain a bisecting GlcNAc structure. Numbers in parentheses indicate the charge state. Source data are available online for this figure.

## Results

### AD patients have higher levels of bisecting GlcNAc on BACE1

We have previously found that a GlcNAc-transferase GnT-III (encoded by *MGAT3*) that generates bisecting GlcNAc is upregulated in AD brains (Akasaka-Manya *et al*, 2010). We also found, using a lectin E4-phytohemagglutinin (PHA) for detection (Cummings & Kornfeld, 1982) (Supplementary Fig S1A and B), that bisecting GlcNAc is mainly expressed in neurons (Supplementary Fig S1C). From these results, we hypothesized that a key molecule involved in AD pathogenesis is modified with this sugar chain to modulate disease progression. We first found a clear mobility shift of BACE1 but not APP in response to GnT-III deficiency (Fig1B), even after the enzymatic removal of O-glycans from APP (Supplementary Fig S1D). We also demonstrated that BACE1 but not APP was recognized by E4-PHA lectin (Fig1B lower panels and Supplementary Fig S1E). The reactivity of E4-PHA to BACE1 was largely absent in GnT-III-deficient (*Mgat3*^−/−^) mice. We confirmed that APP was modified with bisecting GlcNAc in C17 neuroblastoma cells (Akasaka-Manya *et al*, 2008), while APP in the brain was not reactive with E4-PHA (Supplementary Fig S1F), suggesting that modification of APP with bisecting GlcNAc occurs in a limited number of cell types (Kitazume *et al*, 2010) and is non-existent or negligible in the brain. In addition, we found that nicastrin, the only glycosylated subunit of γ-secretase, was slightly modified with bisecting GlcNAc (Supplementary Fig S1G) in spite of the presence of 16 possible *N*-glycosylation sites. In light of these results, as well as several previous reports showing that a change in the glycosylation of nicastrin does not affect γ-secretase activity (Herreman *et al*, 2003; Schedin-Weiss *et al*, 2014), we suggest that BACE1 is a likely functional target of bisecting GlcNAc modification in the Aβ-generation pathway.

The mobility difference in BACE1 disappeared after cleavage of *N*-glycans by peptide:*N*-glycanase (PNGase) F (Fig1C). Furthermore, based on MS/MS analysis of *N*-glycans released from the BACE1 immunopurified from mouse brains (Fig1D; Supplementary Fig S2A–C), we detected diagnostic ions derived from bisecting GlcNAc-containing glycans (Supplementary Fig S2D–H), clearly demonstrating the presence of bisecting GlcNAc on BACE1 *N*-glycans (Fig1D, asterisk). These results indicate that BACE1 is selectively modified with bisecting GlcNAc by GnT-III on its *N*-glycan *in vivo*.

**Figure 2:**
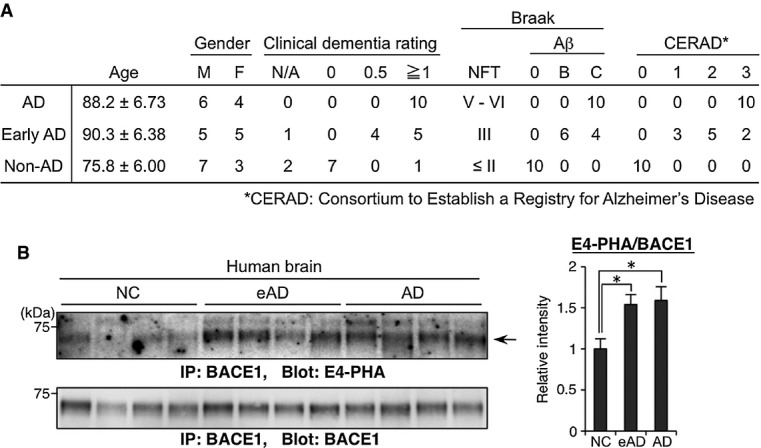
BACE1 is abnormally hyper-modified with bisecting GlcNAc in AD patients

We assumed that the level of bisecting GlcNAc on BACE1 would be increased with disease progression. To test this, BACE1 was immunoprecipitated from the temporal lobe of non-AD, early-stage AD, and late-stage AD patients and then blotted with E4-PHA (Fig2). The lectin reactivity to BACE1 started to increase in early-stage AD. This indicates that the level of bisecting GlcNAc on BACE1 is elevated with disease progression in the human brain, suggesting that this abnormal change in BACE1 glycosylation is involved in AD pathogenesis by modulating β-site cleavage of APP.

Summary of clinical and histological data of non-AD control (NC), early-stage AD (eAD) or AD patients.

BACE1 from temporal lobe membrane fractions of NC, eAD, or AD patients was immunoprecipitated and blotted with E4-PHA (upper) or anti-BACE1 (lower). The signal intensity of E4-PHA relative to that of BACE1 was calculated (*n* = 10). The graph shows means ± SEM (*P < 0.05; one-way ANOVA with *post hoc* Tukey–Kramer test. *P* = 0.014 for NC versus eAD, *P* = 0.028 for NC versus AD). Source data are available online for this figure.

### Loss of bisecting GlcNAc reduces Aβ generation and ameliorates AD pathology in mice

**Figure 3:**
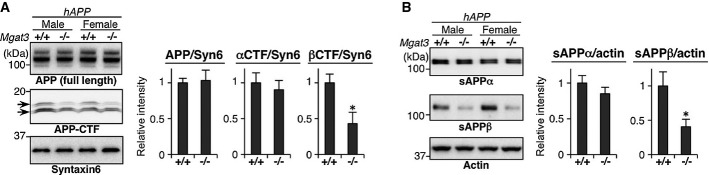
Impaired β-site cleavage of APP in *Mgat3*^−/−^ brain

To investigate how bisecting GlcNAc affects the metabolic pathway of APP *in vivo*, we analyzed APP metabolites in *Mgat3*^−/−^ mice crossed with AD model mice expressing human APP (designated *hAPP*, the APP23 transgenic mouse model for AD). Western blot of APP and its cleaved fragments clearly showed that *hAPP/Mgat3*^−/−^ mice had significantly lower levels of the β-C-terminal fragment of APP (βCTF) and soluble APP cleaved at the β-site (sAPPβ) in their brains than *hAPP/Mgat3*^*+/+*^ mice, whereas the levels of full-length APP, αCTF, and sAPPα were comparable (Fig3), suggesting that bisecting GlcNAc on BACE1 plays a critical role in the β-cleavage process.

APP metabolites from 3-month-old mouse brain membrane (A) or soluble (B) fractions were immunoblotted. The signal intensity was quantified (*n* = 4–5). All graphs show means ± SEM (*P < 0.05; Student's *t*-test for (A) βCTF and (B) sAPPα, and Mann–Whitney *U*-test for the others. *P* = 0.386 for APP, *P* = 0.602 for αCTF, *P* = 0.045 for βCTF, *P* = 0.218 for sAPPα, *P* = 0.022 for sAPPβ). Source data are available online for this figure.

**Figure 4:**
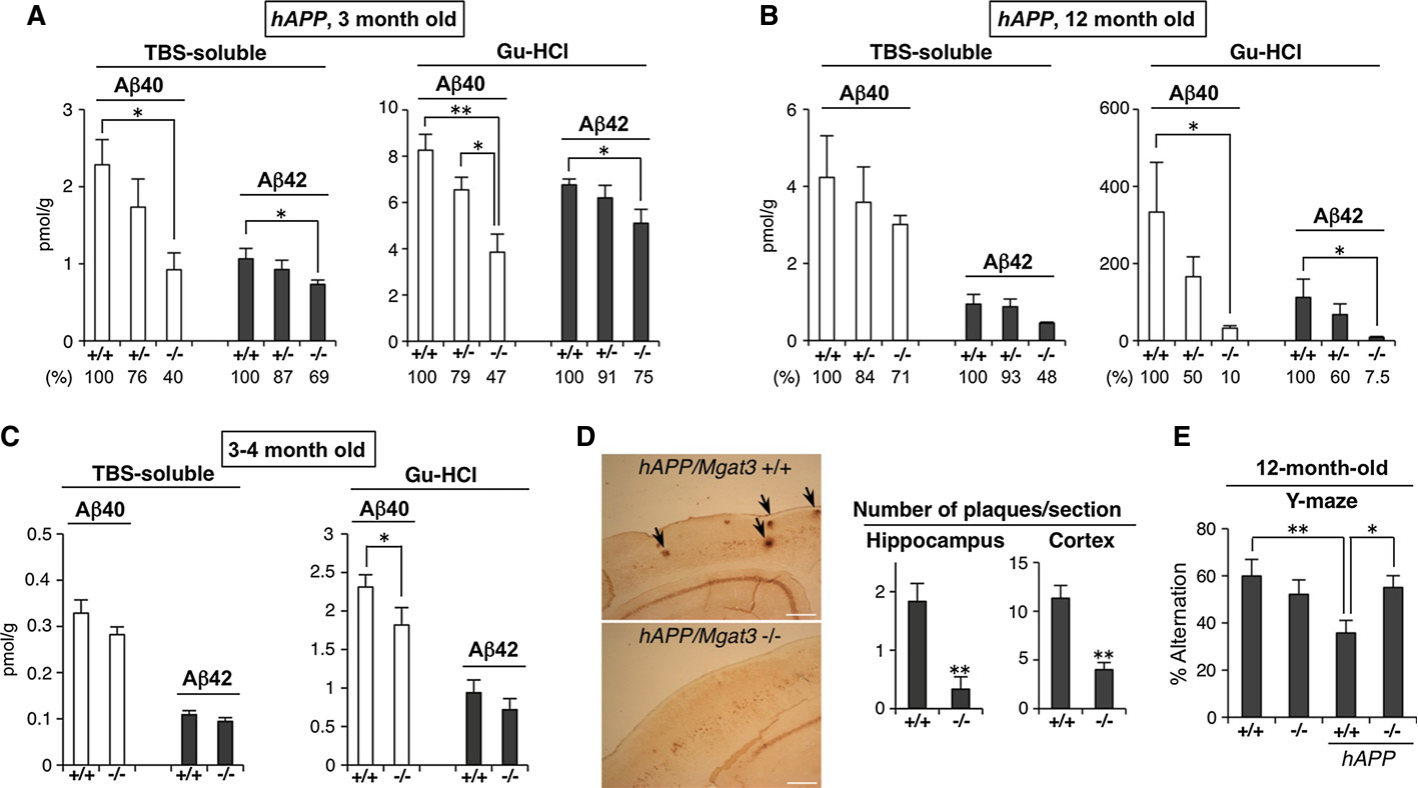
Reduced Aβ load and ameliorated cognitive function in *Mgat3*^−/−^ mouse brain

We next measured the steady-state levels of Aβ40 and Aβ42 (the two major isoforms of Aβ) in 3- and 12-month-old mouse brains. In *hAPP/Mgat3*^*+/+*^ mice, both Aβ40 and Aβ42 levels increased in an age-dependent manner (Fig.4A and B), whereas the amounts of Aβ40/42 in both the Tris-buffered saline (TBS)-soluble and guanidine (Gu)-HCl-extractable fractions were significantly reduced in the *hAPP/Mgat3*^−/−^ mouse brains, consistent with the impairment of β-cleavage by GnT-III deficiency. It should also be noted that a prominent Aβ reduction was observed in 12-month-old mice and that *hAPP/Mgat3*^*+/−*^ mice displayed Aβ levels that were intermediate between those of *hAPP/Mgat3*^*+/+*^ and *hAPP/Mgat3*^−/−^ mice. We also confirmed a slight but significant reduction in Aβ levels in non-APP-transgenic *Mgat3*^−/−^ mice (Fig4C), excluding the possibility that the Aβ reduction by GnT-III deficiency is an APP-transgenic mouse-specific phenomenon. Immunohistochemical analysis revealed that the number of Aβ plaques was markedly decreased in the *hAPP/Mgat3*^−/−^ mice (Fig4D), and the synaptic loss and accumulation of activated astrocytes around Aβ plaques (Saito *et al*, 2014) observed in *hAPP/Mgat3*^*+/+*^ mouse brains were either absent or reduced in *hAPP/Mgat3*^−/−^ brains (Supplementary Fig S3). Moreover, cognitive impairment in *hAPP/Mgat3*^*+/+*^ mice as measured by the Y-maze test was significantly rescued in *hAPP/Mgat3*^−/−^ mice (Fig4E). These results show that deletion of bisecting GlcNAc ameliorates AD-related abnormalities through reduced Aβ deposition caused by impaired β-cleavage.

Amounts of Aβ40 or Aβ42 in the TBS-soluble or Gu-HCl-extractable fraction from (A) 3-month-old or (B) 12-month-old *hAPP/Mgat3*^+/+^, *hAPP/Mgat3*^+/−^, or *hAPP/Mgat3*^−/−^ brains (*n* = 5). *P* = 0.015 for TBS Aβ40, *P* = 0.034 for TBS Aβ42, *P* = 0.001 and 0.012 for Gn–HCl Aβ40, *P* = 0.024 for Gn–HCl Aβ42 in (A), *P* = 0.040 for Gn–HCl Aβ40, *P* = 0.047 for Gn–HCl Aβ42 in (B).

Amounts of Aβ40 or Aβ42 in the TBS-soluble or Gu-HCl-extractable fraction from 3- to 4-month-old *Mgat3*^+/+^ or *Mgat3*^−/−^ brains (*n* = 7–8). An outlier value was rejected by the Smirnov–Grubbs' test (*P* < 0.05). *P* = 0.045 for Gn–HCl Aβ40.

Immunostaining of Aβ plaques in 12-month-old mouse brain (left). Scale bar, 300 μm. The number of FSB-stained Aβ plaques in brain sections prepared from 12-month-old male mice was quantified (right) (*n* = 6). *P* = 0.004 for hippocampus, *P* = 0.0003 for cortex.

The Y-maze test was performed using 12-month-old male *Mgat3*^+/+^, *Mgat3*^−/−^*, hAPP/Mgat3*^+/+^, or *hAPP/Mgat3*^−/−^ mice (*n* = 8–10). **P = 0.004 for *Mgat3*^+/+^ versus *hAPP/Mgat3*^+/+^, *P = 0.022 for *hAPP/Mgat3*^+/+^ versus *hAPP/Mgat3*^−/−^. Data information: All graphs show means ± SEM (*P < 0.05, **P < 0.01. For comparison between two groups, Student's *t*-test was used for (C) and (D) cortex, and Mann–Whitney *U*-test was performed for (D) hippocampus. In other cases, two-way ANOVA with a *post hoc* Tukey–Kramer test (A, B) or the Student–Newman–Keuls test (E) was used.

### BACE1 is more localized to late endosomes/lysosomes, leading to accelerated degradation of BACE1 in Mgat3-deficient mice

**Figure 5:**
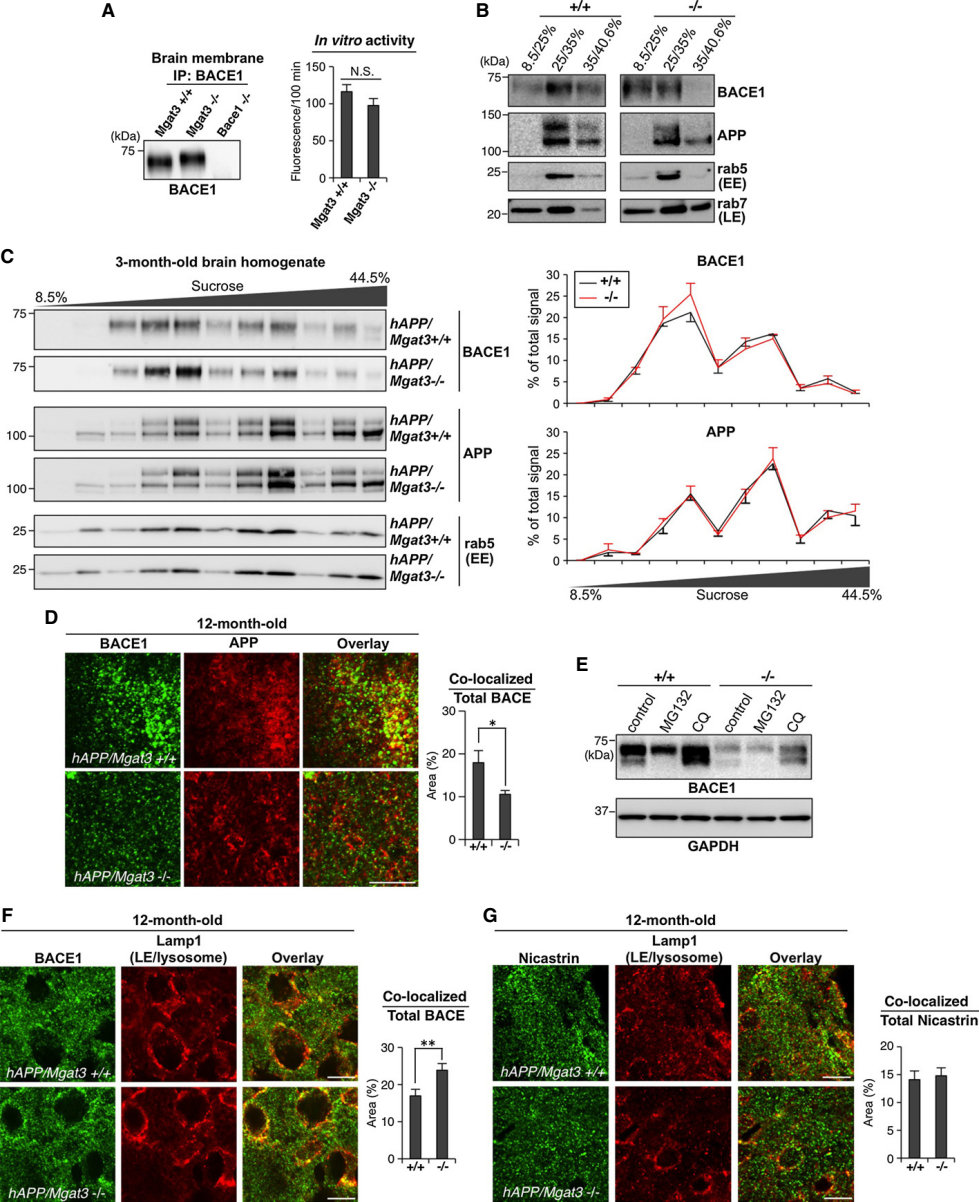
BACE1 is directed to late endosomes/lysosomes in *Mgat3*^−/−^ cells

One possible explanation for the reduced β-cleavage in *hAPP/Mgat3*^−/−^ brain is that bisecting GlcNAc on BACE1 affects its catalytic activity. We therefore measured the *in vitro* enzymatic activity of BACE1 with or without bisecting GlcNAc using fluorescently labeled APP-derived peptide. Immunoprecipitated BACE1 from *Mgat3*^*+/+*^ and *Mgat3*^−/−^ mouse brains showed comparable enzymatic activity *in vitro* (Fig5A). Likewise, overexpression of either GnT-III or dominant negative GnT-III had no effect on the enzymatic activity of recombinant BACE1-Fc (Supplementary Fig S4A and B). In addition, a docking model of the tertiary structure of BACE1 with bisected *N*-glycans showed that all glycans lie apart from the catalytic center (Supplementary Fig S4C). Therefore, it is unlikely that the enzymatic activity of BACE1 is directly modulated by bisecting GlcNAc, although it is still possible that glycans exposed at the molecular surface exert an indirect effect due to the impaired dimerization of BACE1 (Schmechel *et al*, 2004; Westmeyer *et al*, 2004).

BACE1 from 2-week-old mouse brains was immunoprecipitated and immunoblotted (left). Immunoprecipitated BACE1 activity was measured *in vitro* (right, *n* = 3). The values of *Bace1*^−/−^ samples were subtracted as a background. N.S., *P* = 0.101.

MEF homogenates were fractionated by sucrose density centrifugation and immunoblotted for BACE1, APP, rab5, or rab7. EE, early endosome; LE, late endosome.

Brain homogenates were fractionated by sucrose density centrifugation and immunoblotted for BACE1, rab5, or rab9. EE, early endosome; LE, late endosome. Signal intensities were quantified and are shown in the right graphs.

Immunostaining of 12-month-old mouse cerebral cortex for BACE1 and APP. A typical image in the vicinity of plaque-forming area is shown for *hAPP/Mgat3*^+/+^ brain. The area of co-localization was quantified using random images of cerebral cortex (*n* = 10). *P = 0.015.

Immunoblot of BACE1 or GAPDH (loading control) from immortalized MEFs treated with a proteasome inhibitor (MG132) or a lysosome inhibitor (chloroquine; CQ).

Immunostaining of 12-month-old mouse cerebral cortex for BACE1 (F), or nicastrin (G), and Lamp1. LE, late endosome. Scale bar, 10 μm. The area in which co-localized staining was observed was quantified as a percentage of the total BACE1-positive area (right, *n* = 10). **P = 0.007. Data information: All graphs show means ± SEM (Student's *t*-test). Source data are available online for this figure.

We then hypothesized that loss of bisecting GlcNAc results in abnormal subcellular localization of BACE1, leading to the marked reduction in Aβ generation in cells. To test this, we prepared mouse embryonic fibroblasts (MEFs) from *Mgat3*^*+/+*^ and *Mgat3*^−/−^ mice and performed subcellular fractionation of BACE1 and APP by sucrose density gradient centrifugation. We found that BACE1 in *Mgat3*^*+/+*^ MEFs was mainly co-distributed with an early endosome marker and APP, whereas in *Mgat3*^−/−^ cells, BACE1 showed a different distribution from that of APP (Fig5B). This altered localization of BACE1 but not of APP was also found in 3-month-old (Fig5C) and 12-month-old *hAPP/Mgat3*^−/−^ brains (Supplementary Fig S4D), although the difference was less than that observed in *Mgat3*^−/−^MEFs. Immunostaining of mouse brain sections showed that co-localization of BACE1 and APP was significantly reduced in the *hAPP/Mgat3*^−/−^ brain (Fig5D). It was recently reported that BACE1 is localized in endosomal compartments to cleave APP (Das *et al*, 2013), and we confirmed that degradation of BACE1 protein occurs mainly in lysosomes (Koh *et al*, 2005) but not in the proteasome (Fig5E). We therefore expected that, in the absence of bisecting GlcNAc, BACE1 would relocate to late endosomes/lysosomes. Indeed, we found that BACE1 in the brains of *hAPP/Mgat3*^−/−^ mice was more co-localized with the late endosome/lysosome marker Lamp1 than in *hAPP/Mgat3*^*+/+*^ mice (Fig5F). We also stained nicastrin as a control protein and confirmed that co-localization of nicastrin with Lamp1 was not altered in *hAPP/Mgat3*^*+/+*^ mice (Fig5G). Similarly, immunofluorescence staining of *Mgat3*^−/−^ primary neurons revealed increased co-localization of BACE1 but not nicastrin with Lamp1 compared with the pattern in *Mgat3*^*+/+*^ neurons (Supplementary Fig S4E and F). Taken together, these data suggest that the pathological modification of BACE1, bisecting GlcNAc, blocks the lysosomal trafficking of BACE1 in the brain.

**Figure 6:**
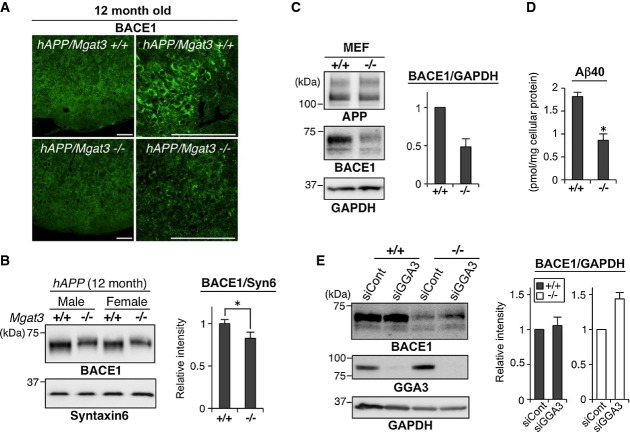
Down-regulation of BACE1 protein in aged *Mgat3*^−/−^ mouse brain

Increased localization of BACE1 to late endosomes/lysosomes in the absence of GnT-III would enhance its lysosomal degradation and lead to down-regulation of BACE1 protein. Although we could not observe a significant reduction in BACE1 protein in 3-month-old *hAPP/Mgat3*^−/−^ mice (Fig1C), immunohistochemical (Fig6A) and Western blot (Fig6B) analyses demonstrated that the level of BACE1 was significantly lower in 12-month-old *hAPP/Mgat3*^−/−^ animals than in age-matched *hAPP/Mgat3*^*+/+*^ mice. Similar down-regulation of BACE1 was also observed in *Mgat3*^−/−^ MEFs as compared with *Mgat3*^+/+^ MEFs (Fig6C), resulting in a significant reduction of Aβ generation in *Mgat3*^−/−^ MEFs (Fig6D).

Immunostaining of 12-month-old brain sections (cerebral cortex) with anti-BACE1. Scale bar, 100 μm.

Proteins from the membrane fractions of 12-month-old mouse brains (*n* = 4–5) were immunoblotted for BACE1, and BACE1 intensity was quantified. *P = 0.044.

Lysates from MEFs were immunoblotted for APP, BACE1, or GAPDH (loading control), and BACE1 intensity was quantified (*n* = 4).

Amount of Aβ40 in the culture medium of immortalized MEFs (*n* = 3). *P = 0.045.

MEFs were transfected with control siRNA or GGA3-siRNA and then immunoblotted for BACE1, GGA3, or GAPDH (loading control) (*n* = 6). Data Information: All graphs show means ± SEM (*P < 0.05; Student's *t*-test for (B) and the Mann–Whitney *U*-test for (D)). Source data are available online for this figure.

Golgi-localized gamma adaptin ear-containing ARF-binding 3 (GGA3) has been reported to promote BACE1 degradation by transporting BACE1 from early endosomes to the late endosome/lysosome pathway (Tesco *et al*, 2007). Knockdown of GGA3 did not affect the level of BACE1 mRNA (Supplementary Fig S4G) but partially rescued the level of BACE1 protein in *Mgat3*^−/−^ MEFs (Fig6E), suggesting that BACE1 in these cells is indeed abnormally targeted to the late endosome/lysosome pathway in a partially GGA3-dependent manner. These findings indicate that GnT-III deficiency causes BACE1 protein to be relocated from early endosomes (where BACE1 is co-localized with APP) to the late endosome/lysosomal pathway partly via GGA3, eventually leading to increased degradation in lysosomes.

### Impaired BACE1 activity in the absence of GnT-III is somewhat selective to APP

**Figure 7:**
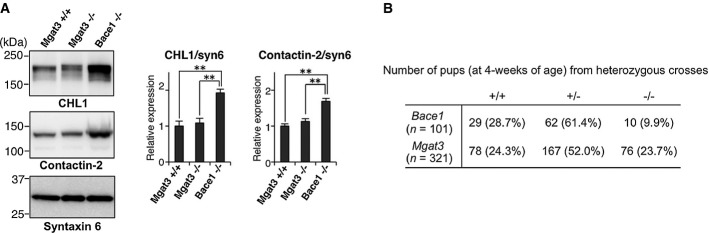
Limited impairment of BACE1 activity in *Mgat3*^−/−^ brain

In addition to APP, BACE1 cleaves several substrate proteins (Hitt *et al*, 2012; Kuhn *et al*, 2012; Vassar *et al*, 2014; Zhou *et al*, 2012), and targeting BACE1 could therefore affect several physiological phenomena via impaired cleavage of these substrates. Indeed, recent studies have reported several abnormalities in *Bace1*^−/−^ mice (Cai *et al*, 2012; Cheret *et al*, 2013; Savonenko *et al*, 2008). Intriguingly, however, the levels of other BACE1 substrates, full-length CHL1, and contactin-2, which were significantly increased in *Bace1*^−/−^ mice due to impaired cleavage, were normal in *Mgat3*^−/−^ mice (Fig7A). This result indicates that the effect of bisecting GlcNAc on BACE1 is somewhat selective to APP. In addition, although a large number of *Bace1*^−/−^ offspring died within 4 weeks after birth (Fig7B) (Dominguez *et al*, 2005), *Mgat3*^*+/−*^ intercrosses produced *Mgat3*^−/−^ mice (23.7%) at normal Mendelian frequency. Moreover, *Mgat3*^−/−^ mice are generally healthy, fertile, and behaviorally normal (Priatel *et al*, 1997). These findings raise the possibility that GnT-III-targeted BACE1 inhibition results in fewer side effects than inhibiting BACE1 itself.

Membrane fractions from 3-week-old *Mgat3*^+/+^, *Mgat3*^−/−^, and *Bace1*^−/−^ mice were immunoblotted for CHL1 (upper), contactin-2 (middle), or syntaxin 6 (lower) (*n* = 4–5). The graphs show means ± SEM (**P < 0.01; two-way ANOVA with *post hoc* Tukey–Kramer test, *P* = 0.006 for *Mgat3*^+/+^ versus *Bace1*^−/−^, *P* = 0.008 for *Mgat3*^−/−^ versus *Bace1*^−/−^ in CHL1/syn6, *P* = 0.001 for *Mgat3*^+/+^ versus *Bace1*^−/−^, *P* = 0.002 for *Mgat3*^−/−^ versus *Bace1*^−/−^ in Contactin-2/syn6).

Number of pups surviving at 4 weeks following a cross between heterozygous male and female mice. Source data are available online for this figure.

## Discussion

**Figure 8:**
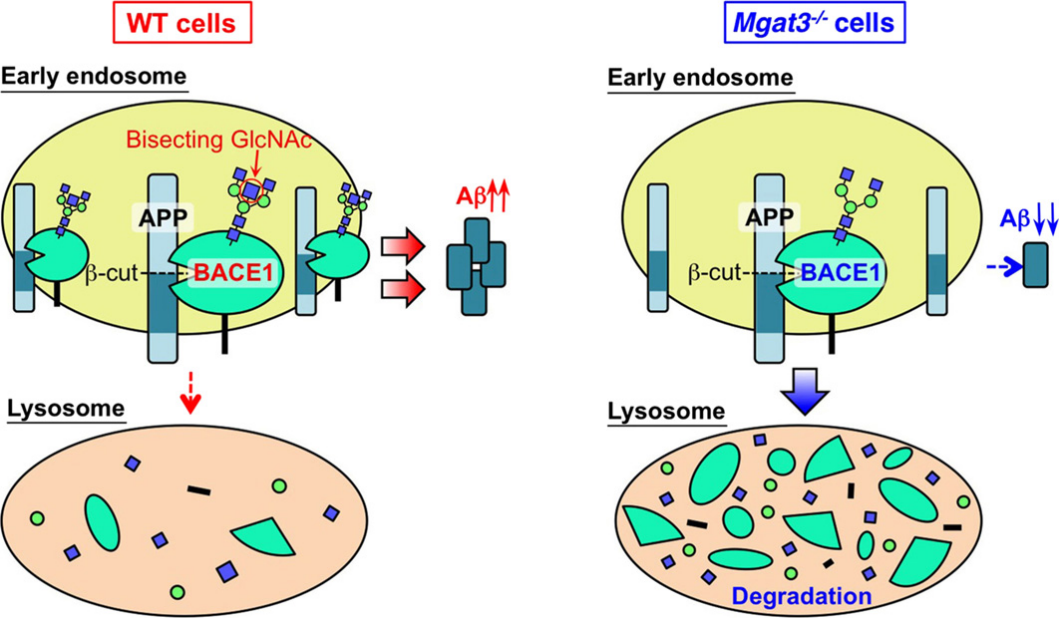
Schematic model of results Lack of bisecting GlcNAc relocates BACE1 from early endosomes (Aβ generation site) to lysosomes, leading to a reduction in both Aβ generation and the level of BACE1 protein.

In this study, we first show that BACE1 is highly modified with bisecting GlcNAc in the brains of AD patients. Our analyses of GnT-III-deficient mice show that lack of bisecting GlcNAc causes a shift in the intracellular localization of BACE1 from early endosomes, where the substrate APP is mainly localized, to late endosomes/lysosomes, thereby enhancing its lysosomal degradation. Both events could contribute to the ameliorated AD-related pathology observed in GnT-III-deficient mice via a significant reduction in Aβ generation (Fig8).

The present study revealed that APP is either not modified or barely modified in the brain (Supplementary Fig S1F), whereas we previously found that it is modified with bisecting GlcNAc in neuroblastoma cells (Akasaka-Manya *et al*, 2008), indicating that bisecting GlcNAc modification on APP occurs in a limited number of cell types. How brain glycoproteins undergo specific glycosylation is another interesting issue.

In addition to Aβ generation, the physiological roles of BACE1 should also be highlighted, given that the use of BACE1 inhibitors as AD therapeutics could disturb these functions. Abnormal phenotypes have been reported in BACE1-deficient mice, including a schizophrenia-like phenotype (Savonenko *et al*, 2008), abnormal muscle spindle formation (Cheret *et al*, 2013), and retinal pathology (Cai *et al*, 2012). Moreover, recent proteomic studies have identified an increased number of novel BACE1 substrates (Kuhn *et al*, 2012; Zhou *et al*, 2012), the biological consequences of whose proteolytic cleavage by BACE1 have not yet been clarified. Unlike BACE1-deficient mice, GnT-III-deficient mice do not display significant abnormalities (Orr *et al*, 2013; Priatel *et al*, 1997), exhibiting only a slight increase in B-220-positive cells and lower vertical activity (Orr *et al*, 2013). Although mutant mice expressing truncated GnT-III have been reported to exhibit several neurological defects such as impaired leg clasp reflex, these abnormalities are considerably milder than those observed in *Bace1*^−/−^ mice and are not seen in GnT-III-deficient mice (Bhattacharyya *et al*, 2002), suggesting that they derive from the presence of truncated GnT-III and not from the loss of full-length GnT-III. These results suggest that inhibiting GnT-III activity would have fewer side effects than the administration of BACE1 inhibitors. Taken together, our findings shed light on the advantages of considering the development of glycan-targeted drugs for AD treatment.

Recent reports have shown that BACE1 is recycled between the Golgi network, the plasma membrane, and endosomes (Tan & Evin, 2012) and that endosomal localization of BACE1 is regulated by GGAs and the retromer, a multiprotein complex required for the recycling of transmembrane proteins from endosomes to the trans-Golgi network. Knockdown of vacuolar protein sorting (Vps) 35, a retromer complex component, results in increased BACE1 localization in endosomes, and *Vps35*^+/−^ mice display increased Aβ generation and deposition (Wen *et al*, 2011). In contrast, the exit of BACE1 from endosomes toward lysosomes is mediated by GGAs, particularly GGA3, which is supported by the finding that *Gga3*^−/−^ brains show increased levels of BACE1 protein (Walker *et al*, 2012). Knockdown of GGA3 partially rescued the instability of BACE1 in *Mgat3*^−/−^ cells, indicating that the absence of bisecting GlcNAc directs BACE1 to the GGA3-mediated lysosomal pathway. It seems that the physical interaction of BACE1 with GGA3 involves the cytoplasmic region of BACE1 and is regulated by BACE1 ubiquitination at the C-terminus (Kang *et al*, 2010), whereas bisected glycans on BACE1 are located in its luminal region, suggesting that an unidentified molecule recognizes bisecting GlcNAc on BACE1 and mediates the interaction between BACE1 and GGA3. We are currently attempting to identify an endogenous lectin-like molecule that displays these properties.

It has been shown that BACE1 is a stress-responsive protease (Kao *et al*, 2004; Vassar *et al*, 2014), and increased BACE1 activity has been observed in AD patients (Yang *et al*, 2003). Our findings demonstrate that BACE1 is highly modified with bisecting GlcNAc in AD patients. Given that bisecting GlcNAc modification is protective for lysosomal degradation of BACE1, we believe that stabilization of BACE1 by this glycan modification also results in enhanced Aβ production in AD patients. Moreover, our finding that BACE1 protein is down-regulated in aged *Mgat3*^−/−^ mice suggests that the regulation of BACE1 by bisecting GlcNAc modification is enhanced with age. Although several studies have previously reported that Aβ-induced stress enhances GnT-III expression in immune cells from AD patients (Fiala *et al*, 2007, 2011), understanding how neuronal GnT-III expression is regulated by oxidative damage or other forms of stress during AD progression remains a topic for future investigation.

## Materials and Methods

### Antibodies and lectin

The commercially available antibodies used were as follows: rabbit anti-BACE1 (5606), anti-nicastrin (9447S), anti-GGA3 (8027), anti-rab5 (3547), anti-rab7 (9367), and anti-rab9 (5118) from Cell Signaling Technology; anti-APP C-term (recognizes C-terminal part of APP, 18961), anti-APP N-term (10D1), and anti-sAPPβ-sw (10321, clone 6A1) from Immuno-Biological Laboratories; anti-Aβ (SIG-39220, clone 4G8) and anti-sAPPα (SIG-39320, clone 6E10) from SIGNET; anti-Lamp1 (ab25630) and anti-PSD95 (ab2723) from Abcam; anti-actin (A4700) from Sigma; anti-syntaxin 6 (610635) from BD Biosciences; anti-GAPDH (MAB374), anti-APP (22C11), and anti-MBP (MAB386) from Millipore; anti-MAP2 (sc-20172) from Santa Cruz Biotechnology; anti-GFAP (13-0300) from Life Technologies; anti-CHL1 (AF2147) and anti-contactin-2 (AF4439) from R&D systems; and anti-Iba1 (019-197419) from Wako. Biotinylated erythroagglutinating phytohemagglutinin (E4-PHA) lectin was from Seikagaku Corporation.

### Mutant mice

The generation of the *Mgat3*-deficient mice, *Bace1*-deficient mice, and transgenic mice expressing human APP with the Swedish mutation (APP23) has been described previously (Luo *et al*, 2001; Priatel *et al*, 1997; Sturchler-Pierrat *et al*, 1997). All mice were from a C57BL/6 genetic background. *Mgat3*-deficient mice were generously provided by Dr. Jamey D. Marth (University of California-Santa Barbara). Mice were housed (3 or fewer mice per cage) at 23 ± 3°C and 55 ± 10% humidity. The light conditions were 14 h : 10 h (lights on at 7:00). All animal experiments were approved by the Animal Experiment Committee of RIKEN.

### Staining of Aβ plaques

For immunostaining, 12-month-old mouse brains (three mice per genotype) were fixed with 4% paraformaldehyde in PBS and embedded in paraffin. Paraffin-embedded coronal sections (5 μm thick) were de-paraffinized according to a standard method and then dipped in 90% formic acid for 5 min. After washing with water (5 min), 0.3% H_2_O_2_ in methanol (30 min), water (10 min), and PBS (3 min), the sections were blocked with 3% BSA in PBS for 30 min, followed by overnight incubation with the primary antibody (1:200 dilution; 4G8). The sections were then incubated with biotinylated anti-mouse IgG, followed by HRP-avidin using the VECTASTAIN ABC standard kit (Vector Laboratories). Signals were visualized by DAB staining. For quantification of the number of Aβ plaques, frozen sections (30 μm thick) from 12-month-old male mice (six mice per genotype) were incubated with 0.05% (w/v) FSB (Dojindo) in EtOH/PBS (1/1, v/v) for 30 min at room temperature and then washed three times with EtOH/PBS (1/1). Fluorescence was visualized using an Olympus FV-1000 confocal microscope.

### Preparation of membrane and soluble fractions from mouse brain

Brains were homogenized with seven volumes of TBS containing a protease inhibitor cocktail (Roche) using a Potter-style tissue grinder. Homogenates were ultracentrifuged at 100,000 × *g* for 30 min at 4°C, and the resultant pellet and supernatant were used as the membrane and soluble fractions, respectively.

### Glycosidase treatment

For PNGase F treatment, proteins (50 μg) were denatured by boiling in 20 μl of PBS containing 0.5% SDS, 1% 2-mercaptoethanol, and 4 mM EDTA. After the solution had been diluted with four volumes of PBS containing Nonidet P-40 (NP-40, final concentration 0.5%), PNGase F (1,000 units, New England Biolabs) was added, and the solution was incubated for 3 h at 37°C. For sialidase and *O*-glycosidase treatment, APP was first immunoprecipitated from mouse brain membrane fraction. After washing the beads with TBS/0.1% NP-40, immobilized APP was incubated with 10 mU sialidase (*Arthrobacter ureafaciens*, Nacalai tesque) with or without 1.5 mU *O*-glycosidase (Roche) in acetate buffer (100 mM sodium acetate pH 4.5, 50 mM NaCl, 0.1% NP-40, 1 mM EDTA, protease inhibitor cocktail) for 12 h at 37°C.

### Lectin pulldown and immunoprecipitation

For lectin pulldown, the membrane fraction (obtained from 250 μl of homogenate) was solubilized with 750 μl of TBS containing 1% Triton X-100 and a protease inhibitor cocktail (Roche) and then ultracentrifuged at 100,000 × *g* for 15 min. The supernatant was incubated with 100 μl of E4-PHA-agarose (Seikagaku Corporation) for 2 h at 4°C. The beads were washed twice with an excess volume of TBS containing 0.1% Triton X-100, and the bound proteins were eluted with SDS sample buffer. For immunoprecipitation, the membrane fraction (obtained from 250 μl of homogenate) or cell pellet (obtained from a 10-cm dish) was lysed with TBS (750 μl for the brain membrane fraction and 500 μl for the cell pellet) containing 0.5% NP-40 and protease inhibitor cocktails and then ultracentrifuged at 100,000 × *g* for 15 min. The supernatant was incubated with antibody (3–5 μg) for 10 min at 4°C, after which protein G-Sepharose 4 Fast Flow (20 μl, GE Healthcare) was added to the mixture, followed by rotation for 2 h at 4°C. The beads were washed three times with TBS containing 0.1% NP-40, and bound proteins were eluted with SDS sample buffer.

### Y-maze test

The Y-maze test was performed using 12-month-old male mice as described previously with minor modifications (Saito *et al*, 2011). Tests were performed at a light intensity of 90 lx at the level of the platform.

### Western and lectin blotting

Proteins were separated by 4–20% gradient SDS–PAGE and then transferred to PVDF or nitrocellulose membranes. After incubation with 5% non-fat dried milk in TBS containing 0.1% Tween-20, the membranes were incubated with primary antibody, followed by HRP-conjugated secondary antibody. Signals were detected with SuperSignal West Dura Extended Duration Substrate (Thermo Scientific) using ImageQuant LAS-4000mini (GE Healthcare). For lectin blotting, nitrocellulose membranes were blocked with TBS containing 0.1% Tween-20 for 30 min at room temperature. The membranes were then incubated with biotinylated E4-PHA lectin (1:500) that had been diluted with TBS containing 0.1% Tween-20, followed by incubation with HRP-avidin (VECTASTAIN ABC Standard Kit). The intensity of the resultant protein bands was quantified using ImageQuant TL software (GE Healthcare). For quantification of the levels of APP metabolites and BACE1, proteins from the brains of 3-month-old (three male and two female) and 12-month-old (two male and three female) mice were analyzed. Each set of experiments was repeated at least three times to confirm the results.

### Aβ ELISA

Aβ ELISA was performed as described previously (Iwata *et al*, 2004; Saito *et al*, 2011) with slight modifications. Brains from 3-month-old (three male and two female) and 12-month-old (two male and three female) *hAPP* mice or from 3- to 4-month-old (six male and two female) non-*hAPP* mice were homogenized with seven volumes of TBS containing a protease inhibitor cocktail (Roche) using a Potter-style tissue grinder. Homogenates were ultracentrifuged at 100,000 × *g* for 30 min at 4°C, and the resultant supernatant was used as a TBS-soluble fractions. The pellet was then homogenized again in TBS and ultracentrifuged at 100,000 × *g* for 10 min at 4°C. The resultant pellet containing insoluble and membrane-associated Aβ was suspended in 10 volumes of buffer (50 mM Tris–HCl, 6 M guanidine, protease inhibitor cocktail, pH 7.6) and sonicated. After incubation for 1 h at room temperature, a Gu-HCl-extracted fraction was obtained by ultracentrifugation at 100,000 × *g* for 20 min at 25°C. For ELISA, the Gu-HCl fraction was diluted 12-fold with phosphate buffer, and a 1/11 volume of 6M Gu-HCl was added to the soluble fraction to normalize the effect of Gu-HCl. ELISA was carried out using the Human/Rat βamyloid (40) ELISA kit and Human/Rat βamyloid (42) ELISA kit High-Sensitive (Wako). The values in *Bace1*^−/−^ mice were subtracted as a background. In the case of MEFs, cells were cultured with complete culture medium (DMEM supplemented with 10% fetal bovine serum [FBS]) for 24 h, after which the culture medium was collected. Following centrifugation at 10,000 × *g*, the supernatants were directly analyzed by ELISA without dilution. Cells were also collected for protein measurements.

### Glycan analysis of BACE1 from mouse brain

One hundred brains from 1-week-old mice were homogenized in 200 ml of buffer (TBS containing a protease inhibitor cocktail) and then centrifuged at 500 × *g* for 10 min to remove the nuclei of the cells. The supernatant was centrifuged at 105,000 × *g* for 2 h, after which the pellet was lysed with buffer (TBS containing 0.5% NP-40 and a protease inhibitor cocktail), followed by centrifugation at 105,000 × *g* for 2 h. 15 mg of Dynabeads protein G (Life Technologies) was added, followed by 30 min rotation to remove the IgG in the sample. The beads were then removed, and 240 μg of rabbit anti-BACE1 (5606, Cell Signaling Technology) and 40 mg Dyna-beads protein G were added. After 60 h of rotation, the beads were collected and washed three times with an excess volume of TBS containing 0.1% NP-40. They were then further washed with 80 μg of BACE1 C-terminal peptide (CLRQQHDDFADDISLLK, 200 μg/ml in TBS 0.1% NP-40) to remove weakly bound proteins, followed by TBS containing 0.1% NP-40. Proteins bound to the beads were eluted by 800 μl of the buffer (50 mM glycine-HCl, pH 2.5), followed by immediate neutralization by adding 18 μl of 1 M Tris–HCl pH 8.5. The solvent was evaporated by SpeedVac, and the proteins were dissolved and separated by SDS–PAGE. After transfer to PDVF membrane and protein staining with Direct Blue, the band corresponding to BACE1 or the IgG heavy chain was excised. *N*-glycans from these glycoproteins were released and analyzed as described previously (Nakano *et al*, 2011) with some modifications. After release and reduction, *N*-glycans were desialylated by incubating with 2 M acetic acid at 80°C for 2 h. *N*-glycan alditols were separated on a carbon column (5 μm HyperCarb, 1 mm I.D. × 100 mm, Thermo Fisher Scientific) using an Accela HPLC pump (flow rate: 50 μl/min). The eluate was continuously introduced into an ESI source (LTQ Orbitrap XL, Thermo Fisher Scientific). MS spectra were obtained in the negative ion mode using Orbitrap MS (mass range m/z 500 to m/z 2,500), and MS/MS spectra were obtained using Iontrap MS. Monoisotopic masses were assigned with possible monosaccharide compositions using the GlycoMod software tool (mass tolerance for precursor ions is ±0.005 Da).

### *In vitro* BACE1 activity assay

Native BACE1 was extracted from the membrane fraction (obtained from 250 μl of homogenate) of 2-week-old mouse brain tissue with TBS containing 1% Triton X-100 and a protease inhibitor cocktail. After centrifugation at 100,000 × *g*, the supernatant was subjected to overnight immunoprecipitation with 5 μg of anti-BACE1 antibody and 20 μl of protein G-Sepharose. The beads were washed twice with TBS containing 0.2% Triton X-100 and used directly as an enzyme source. For preparation of recombinant BACE1-Fc, COS-7 cells were co-transfected with pEF-Fc/BACE1, together with pCXN2, pCXN2/GnT-III, or pCXN2/GnT-III D319A. After 6 h, the medium was replaced with Opti-MEM I followed by further culture for 3 days. Recombinant BACE1 proteins (0.75 μg of each protein), purified through a protein G column, were used as an enzyme source. Assays were performed in a solution containing 100 mM sodium acetate buffer at pH 4.5, 20 μM substrate (3212v, Peptide Institute Inc.), 0.25% Triton X-100, protease inhibitor cocktails, and an enzyme source in a final volume of 50 μl, followed by incubation for 100 min at 37°C. We confirmed that for an incubation period up to 120 min, BACE1 hydrolyzes the substrate in a time- and dose-dependent manner. The reaction was stopped by adding 50 μl of denaturing buffer (1% SDS, 100 mM Tris–HCl pH 9.5), and fluorescence was measured using a Wallac 1420 ARVOsx multilabel counter (Perkin Elmer) at excitation and emission wavelengths of 340 nm and 405 nm, respectively.

### Plasmids

The construction of pEF-Fc/human BACE1 (soluble BACE1-Fc) has been described previously (Kitazume *et al*, 2001). pCXN2/human GnT-III was constructed as described previously (Kitada *et al*, 2001). pCXN2/human GnT-III D319A (dominant negative form (Ihara *et al*, 2002)) was constructed using a QuickChange XL Site-Directed Mutagenesis Kit (Agilent Technologies) with primers (GTCTTCATCATTGACGATGCGGCCGAGATCCCGGCCCGTGACG and its complementary sequence). pLenti6/human BACE1 was constructed using PCR to amplify the fragment encoding full-length BACE1 with primers (AGGGAATTCGCCACCATGGCCCAAGCCCTGCCCTG and TCCTCACTTCAGCAGGGAGATGT). The fragment was digested with EcoRI and then inserted into pLenti6/V5-GW/LacZ which had been digested with EcoRI and EcoRV. The plasmid encoding SV40 large T antigen was kindly provided by Dr. Jianguo Gu (Tohoku Pharmaceutical University).

### Immunofluorescence staining

To prepare frozen brain sections, mice were transcardially perfused with PBS followed by 4% paraformaldehyde in PBS. Brains were sequentially immersed in the same fixative for 16 h and 30% sucrose in PBS for 3 days (with daily renewal of the buffer) at 4°C. Brain sections (30 μm thick) were stained using the floating method. Briefly, sections were incubated with PBS containing 50 μg/ml digitonin and 3% BSA for 20 min at room temperature, followed by incubation with primary antibody or biotinylated lectin (overnight at 4°C) and Alexa-labeled secondary antibody or streptavidin (30 min at room temperature). For double staining of Aβ plaques, sections were first stained with FSB as described above (see ‘Staining of Aβ plaques’) and then stained with antibodies. Fluorescence was visualized using an Olympus FV-1000 confocal microscope, with data acquisition and quantification of the signals or co-localized area being carried out using FV10-ASW ver.1.7 software (Olympus).

### Preparation of mouse embryonic fibroblasts (MEFs)

Male and female *Mgat3*^*+/−*^ mice were mated to obtain E13 littermate embryos. After the head and liver were removed from each embryo, the remaining tissues were minced. The cells from each embryo were then incubated at 37°C for 30 min in 5 ml of PBS containing 0.05% trypsin, 0.53 mM EDTA, and 0.004% DNase I. After the cells were collected by centrifugation, they were resuspended and incubated twice for 30 min in the same buffer. They were then suspended in complete culture medium (DMEM supplemented with 10% FBS) before being passed through a 100-μm cell strainer. After centrifugation at 270 × *g* for 5 min at 4°C, the cells were resuspended in the culture medium and plated on a 15-cm dish (one dish per embryo). Genotyping of each embryo was carried out using tissue pieces with the primers described elsewhere (Priatel *et al*, 1997). The MEFs were immortalized by transfection with a plasmid encoding SV40 large T antigen, after which the transformed cells were selected with 400 μg/ml Zeocin.

### Preparation of primary neurons

Wild-type (or *Mgat3*^−/−^) male and female mice were crossed, and embryos were used at E16–18. Embryonic brains were minced in Neurobasal medium (Gibco) and then incubated for 5 min on ice. After removing the supernatant, 5 ml of Neurobasal medium was added, followed by a further 5-min incubation on ice. The cells were then incubated in Hanks' balanced salt solutions (HBSS, Gibco) containing 0.25% trypsin for 15 min at 37°C. DNase I (final 0.05%) was added, and the cells were incubated for 1 min at 37°C. After pipetting ten times and centrifugation, the cell pellet was suspended and incubated in HBSS containing 0.05% DNase I for 3 min at 37°C. The cells were centrifuged and resuspended in complete culture medium (Neurobasal medium supplemented with 2% B27 and 0.5 mM glutamine) and passed through a 100-μm strainer. They were then seeded at 5 × 10^5^ or 2.5 × 10^5^/ml per dish or chamber slide which had been precoated with poly-D-lysine. After 2 days, Ara-C (C6645, Sigma) was added at 5 μM to kill dividing non-neuronal cells.

### Lentiviral infection

To produce the lentiviral vector, 293FT cells plated in 10-cm dishes were simultaneously transfected with 2.25 μg of pLP1, 2.25 μg of pLP2, 4.5 μg of pLP/VSVG, and 3 μg of pLenti6/human BACE1 using Lipofectamine 2000. The following day, the medium was replaced with fresh medium, and after 48 h (72 h post-transfection), the medium was collected. Cells and debris were removed by centrifugation, and the medium was passed through a filter (pore size 0.45 μm). The viruses were collected by centrifugation at 50,000 × *g* for 2 h at 20°C and then resuspended in Neurobasal medium to concentrate the viruses (12-fold). The virus solution was directly added to the primary neuron culture medium after 2 days *in vitro*.

### Cell culture and transfection

C17, COS-7, and MEF cells were cultured in DMEM supplemented with 10% FBS. For plasmid transfection, cells at 80% confluency on a 10-cm dish were transfected with 4–8 μg of each plasmid using 10–20 μl of Lipofectamine 2000 (for COS-7 cells).

### siRNA treatment

For knockdown experiments, FlexiTube siRNAs (Qiagen) were used. MEFs at 30% confluency on 10-cm (or 6-cm) dishes were transfected with 200 pmol (or 80 pmol) of control siRNA (AllStars negative control siRNA, Qiagen) or siRNA for GGA3 (SI01011451) using 20 μl (or 8 μl) of Lipofectamine 2000. At 24 h or 48 h after transfection, cells were used for other experiments.

### Subcellular fractionation by sucrose density gradient centrifugation

Subcellular fractionation was performed as described previously (Aniento *et al*, 1993) with modifications. All the buffers contained 20 mM Tris–HCl pH 7.5 and 3 mM imidazole. Cells were homogenized with buffer containing 8.5% sucrose and protease inhibitor cocktail by passaging 10 times through a 26-gauge needle. After removal of nuclei and debris by centrifugation at 1,000 × *g* for 5 min, the concentration of sucrose was adjusted to 40.6%. The sample (1 ml) was loaded at the bottom of a tube and overlaid with 1 ml of 35% sucrose, 1 ml of 25% sucrose, and 1 ml of the homogenization buffer. After ultracentrifugation using an S52ST rotor at 150,000 × *g* for 90 min, interfaces at 8.5/25, 25/35, and 35/40.6% were recovered. To sediment membranes, the collected samples were diluted fourfold and then ultracentrifuged at 180,000 × *g* for 30 min. The resultant pellets were solubilized, and protein concentrations were measured. An equivalent amount of protein was taken from each fraction for Western blotting. In the case of mouse brains, the whole brain was first homogenized with seven volumes of the same buffer containing 8.5% sucrose, after which the nuclei and debris were removed. The postnuclear solution was adjusted to 40.6% sucrose, and 1 ml of the sample was loaded at the tube and overlaid with 1 ml of 35% sucrose, 1 ml of 30% sucrose, and 1 ml of 25% sucrose solutions. After ultracentrifugation using a S52ST rotor at 100,000 × *g* for 90 min, the top fraction and interfaces at 25/30, 30/35, and 35/40.6% were collected. For separation of 11 fractions, a previously described method (Tan *et al*, 2013) was modified. 1 ml of 44.5% sucrose was loaded in the tube and overlaid with 3 ml of 39.7% sucrose, 3 ml of 34.2% sucrose, 3 ml of 27.4% sucrose, and 1 ml of the postnuclear solution. After ultracentrifugation using P40ST rotor at 120,000 × *g* for 16 h, each fraction (1 ml) was collected. Equal volumes of the fractions were used for Western blotting.

### RNA extraction, reverse-transcription and quantitative PCR

Total RNA from cultured cells was extracted using TRIzol (Invitrogen). One microgram of total RNA was reverse-transcribed using the SuperScript III First-Strand Synthesis System (Invitrogen) with random hexamers. For BACE1 primers and probe, we used Assays-on-Demand gene expression products, and cDNAs were added to the TaqMan Universal PCR Master Mix (Applied Biosystems). The probe for BACE1 was labeled with FAM at its 5′-end and with the quencher MGB at its 3′-end. The probes for rRNA were labeled with VIC at their 5′-end and with the quencher TAMRA at their 3′-end. The cDNAs were amplified using an ABI PRISM 7900HT sequence detection system (Applied Biosystems). The level of BACE1 mRNA was measured in duplicate and normalized to the corresponding rRNA level.

### Human samples

The clinical study was approved by the ethical committees of RIKEN, Tokyo Metropolitan Institute of Gerontology, and Tokyo Metropolitan Geriatric Hospital. Frozen tissues from postmortem brain were obtained from the Brain Bank for Aging Research, which consists of consecutive autopsy cases from a general geriatric hospital with informed consent obtained from the relatives for each autopsy. Handling of the brains and diagnostic criteria have been described previously (Akasaka-Manya *et al*, 2010). One gram of temporal pole tissue was sampled from 10 cases each with AD or early AD, and age-matched controls (the same cases as reported in the previous study(Akasaka-Manya *et al*, 2010)). A summary of the clinical and histological data is shown in Fig2A. The brains were homogenized with five volumes of buffer (20 mM Tris–HCl, pH 7.4, 150 mM NaCl, 5 mM EDTA, and protease inhibitor cocktail) using stainless steel beads (7.9 mm) in Micro Smash (TOMY) for 40 (1^st^) and 20 (2^nd^) seconds at 3,500 rpm. The homogenates were ultracentrifuged at 100,000 × *g* for 1 h to obtain membrane fractions. BACE1 immunoprecipitation and Western blotting were carried out (no blinding) as described above for mouse brain with slight modifications. We used magnetic Dynabeads protein G for protein pulldown. The lysates were first pre-cleared by the beads in the absence of antibody addition.

### Construction of a three-dimensional model of *N*-glycosylated human BACE1

A 3D structural model of human BACE1 with bisected *N*-glycans was generated by GlyProt (Bohne-Lang & von der Lieth, 2005). The atomic coordinate of unglycosylated human BACE1 (PDB code: 2qp8) was used for the construction of the glycosylated model (Shimizu *et al*, 2008), and the bisected *N*-glycans (GlcNAc_1_Man_3_GlcNAc_2_) were attached to four *N*-glycosylation sites, Asn153, Asn172, Asn223, and Asn354.

### Statistical analysis

All data are shown as mean ± SEM. For comparison of the means between two groups, statistical analysis was performed by applying an unpaired one-sided Student's *t*-test after confirming equality between two groups and normality by a Kolmogorov–Smirnov test. If these tests were not passed, a Mann–Whitney *U*-test was performed. Comparisons of the means among more than two groups were done by a one-way or two-way analysis of variance (ANOVA) followed by a *post hoc* test, in which a Student–Newman–Keuls test (SigmaPlot software, ver.11; Systat Software Inc.) or Tukey–Kramer test was applied. *P*-values < 0.05 were considered to be significant.

## The paper explained

### Problem

Alzheimer's disease (AD) is the most common dementia and is a serious issue in aging populations throughout the world. However, efforts to develop therapeutic agents for AD have so far achieved limited success. The deposition of amyloid β (Aβ) peptide in the brain, resulting in Aβ plaques, is the biochemical hallmark of AD. Currently, one of the most promising targets for AD therapy is the enzyme BACE1 (β-site amyloid precursor protein cleaving enzyme-1), which generates Aβ from its precursor protein. Several BACE1 inhibitors are in clinical trials, but there is a concern about side effects, given that BACE1 also cleaves other proteins in the brain. Indeed, *Bace1* knockout mice exhibit severe phenotypes such as schizophrenia-like symptoms, muscle abnormality, retinal pathology, and early lethality. Therefore, modulation of BACE1 function with fewer detrimental side effects would be a rational and promising strategy to ameliorate AD pathology. However, little is known about the regulation of BACE1 function at the level of cellular expression and compartmental localization during health and disease.

### Results

In this study, we show that a unique sugar modification, ‘bisecting GlcNAc’, on BACE1 is a novel regulator of cellular BACE1 stability. We found that BACE1 is abnormally modified, with higher levels of bisecting GlcNAc being observed in AD patients, suggesting the pathological involvement of this sugar modification on BACE1 during AD development. Using AD model mice and knockout mice lacking the glycosyl enzyme (GnT-III) responsible for the biosynthesis of bisecting GlcNAc, we demonstrated that the loss of bisecting GlcNAc diminishes Aβ plaque formation by reducing BACE1-mediated Aβ generation. The decrease in Aβ generation in the knockout mice was caused by a shift in the intracellular distribution of BACE1 from early endosomes (where Aβ precursor protein is localized) to late endosomes/lysosomes, leading to faster lysosomal degradation of BACE1. These results indicate that bisecting GlcNAc is a novel pathological modification of BACE1 which delays its degradation.

### Impact

Knockout mice for bisecting GlcNAc exhibit a robust reduction in Aβ production but show almost no abnormality in contrast to the severe phenotypes of *Bace1* knockout mice. The latter phenotypes are caused by the impaired cleavage of physiological BACE1 substrates, whereas we found that a BACE1 substrate other than Aβ-precursor protein is normally cleaved in GnT-III-deficient mice, suggesting that the inhibitory effects of bisecting GlcNAc on BACE1 are selective to the Aβ-generation pathway. Therefore, inhibiting the biosynthesis of bisecting GlcNAc could block the AD-related pathological effect of BACE1, while having only weak detrimental effects on normal physiological BACE1 functions. These findings suggest that the biosynthetic pathway for bisecting GlcNAc could serve as a novel and promising drug target for AD therapy, resulting in fewer side effects than BACE1 inhibitors.

## Supporting Information

Supplementary Figure Legends

Review Process File
