# Supplementary figures and images for: An aberrant sugar modification of BACE1 blocks its lysosomal targeting in Alzheimer's disease
Source: EMBO Mol Med. 2015 Jan 15;7(2):175–89. doi: 10.15252/emmm.201404438 (PMC4328647; doi:10.15252/emmm.201404438)

Figure S1

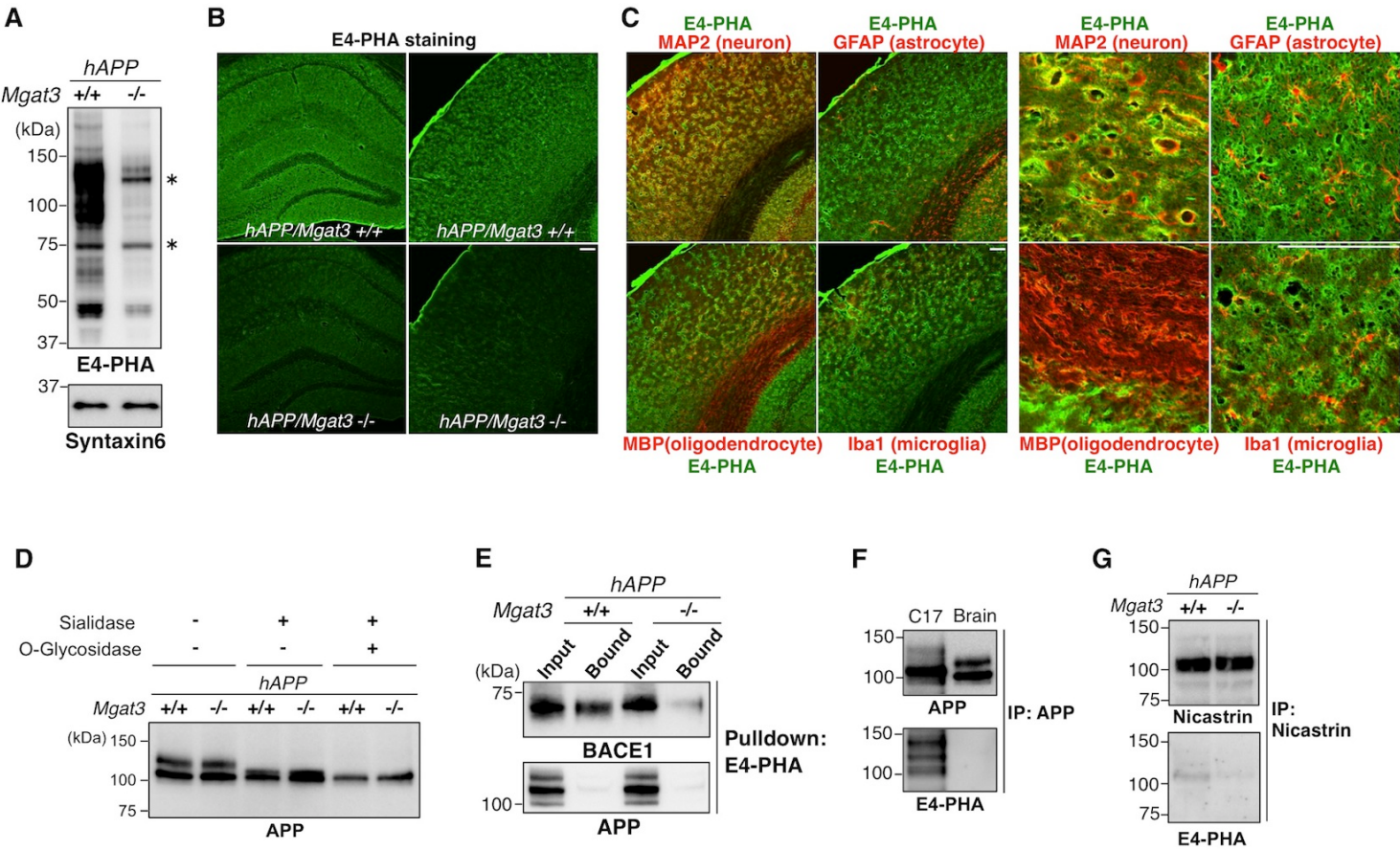

Figure S2

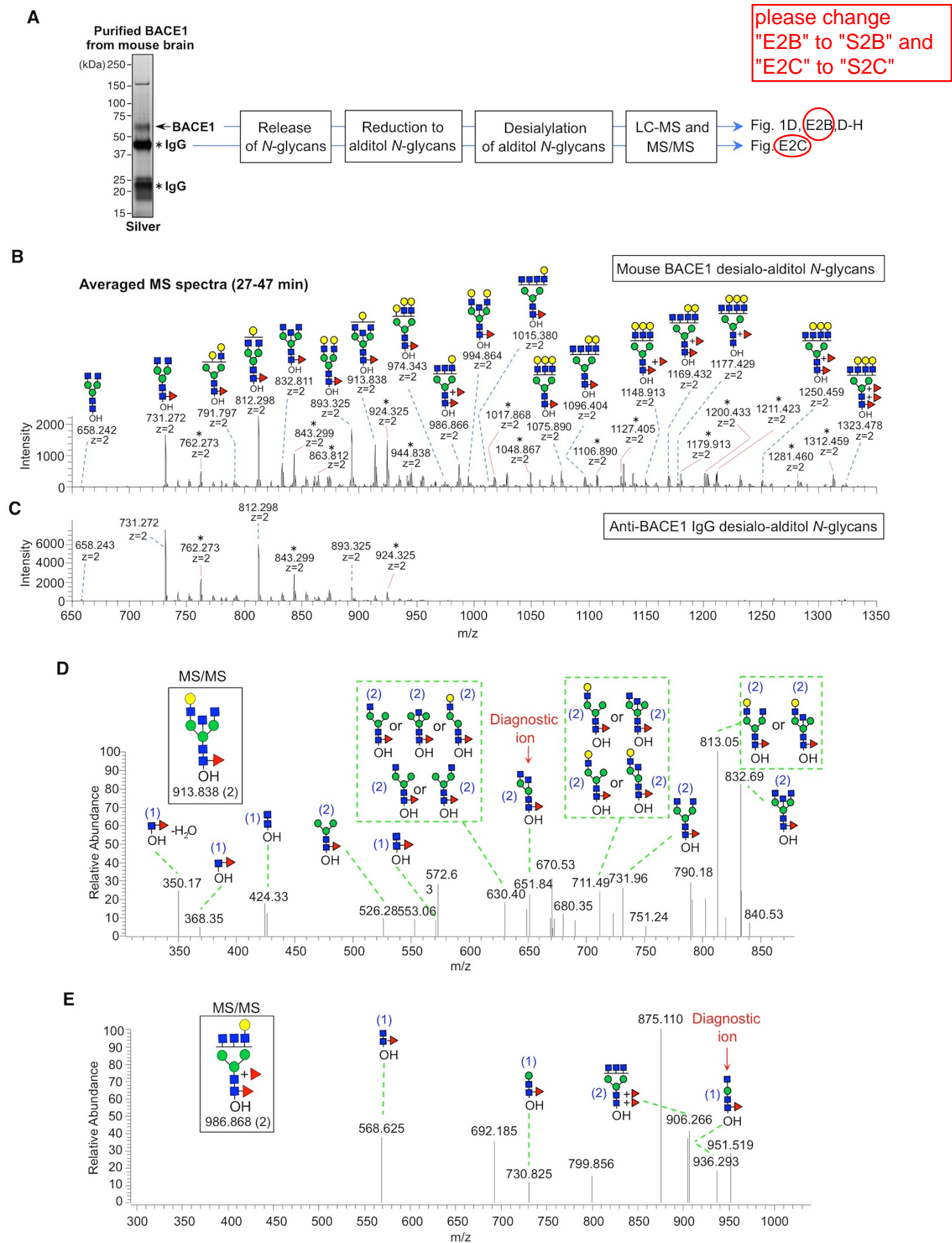

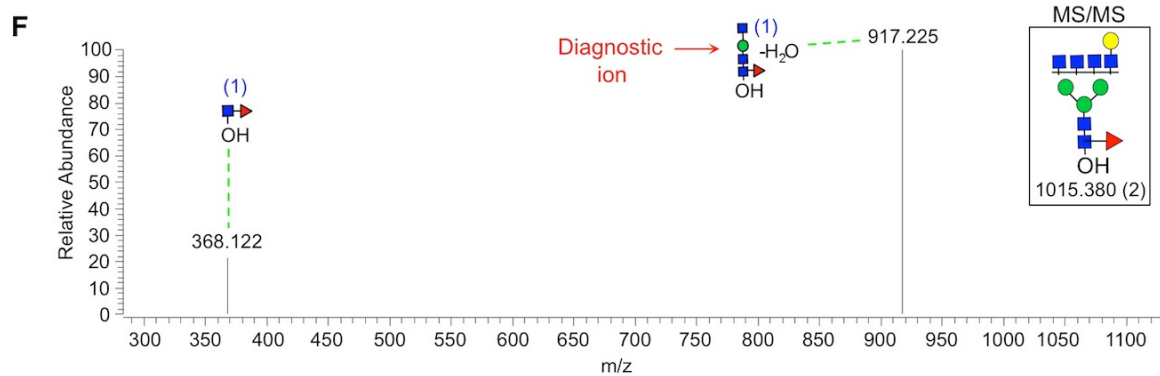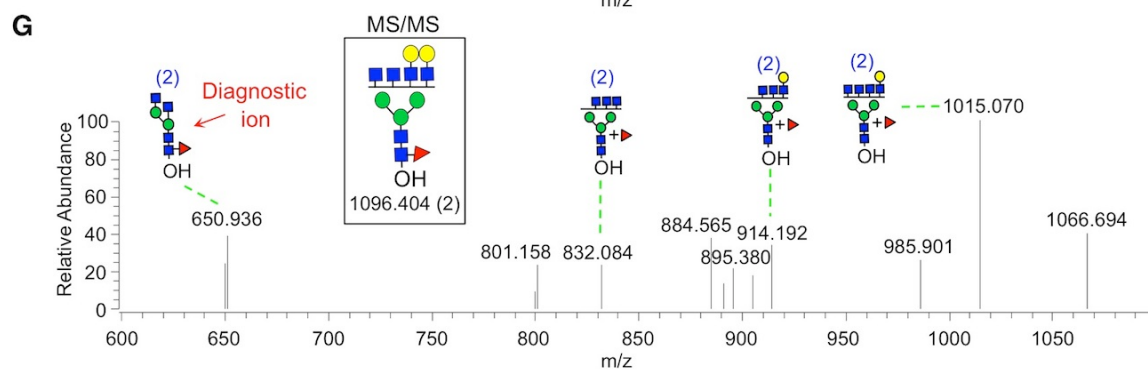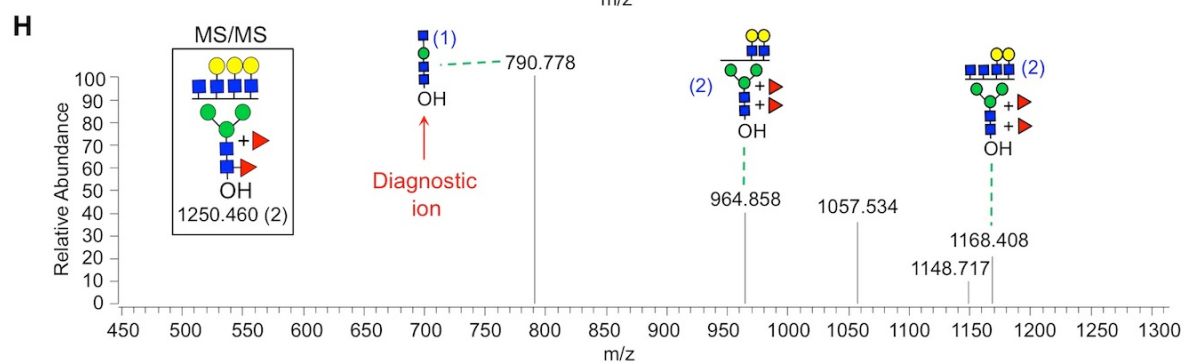

Figure S3

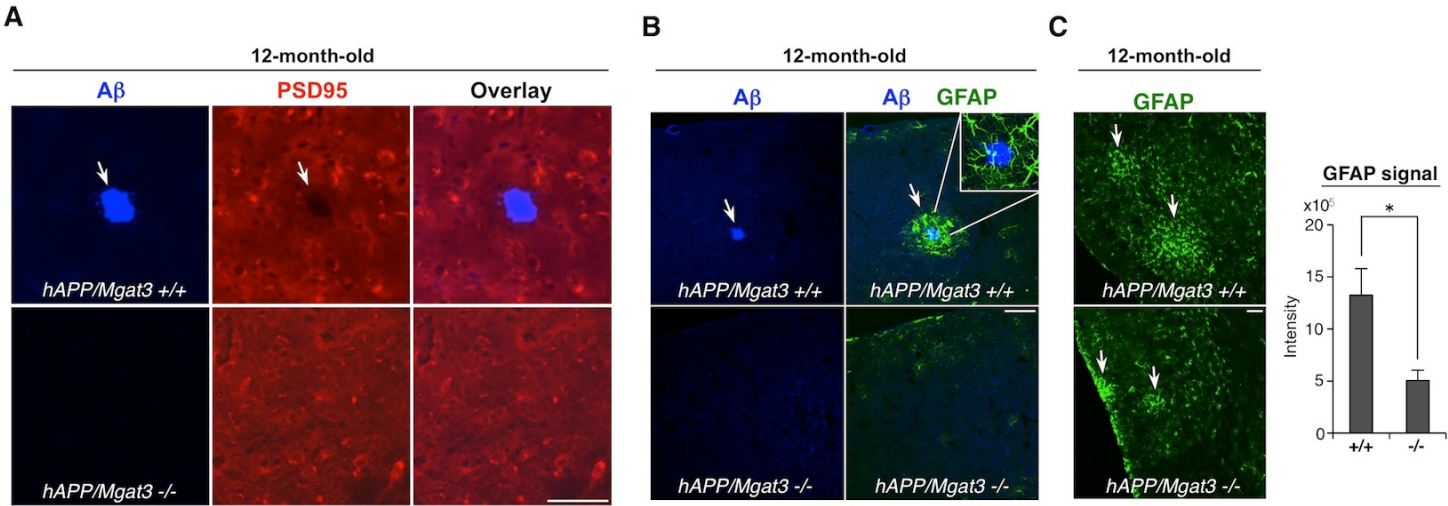

Figure S4

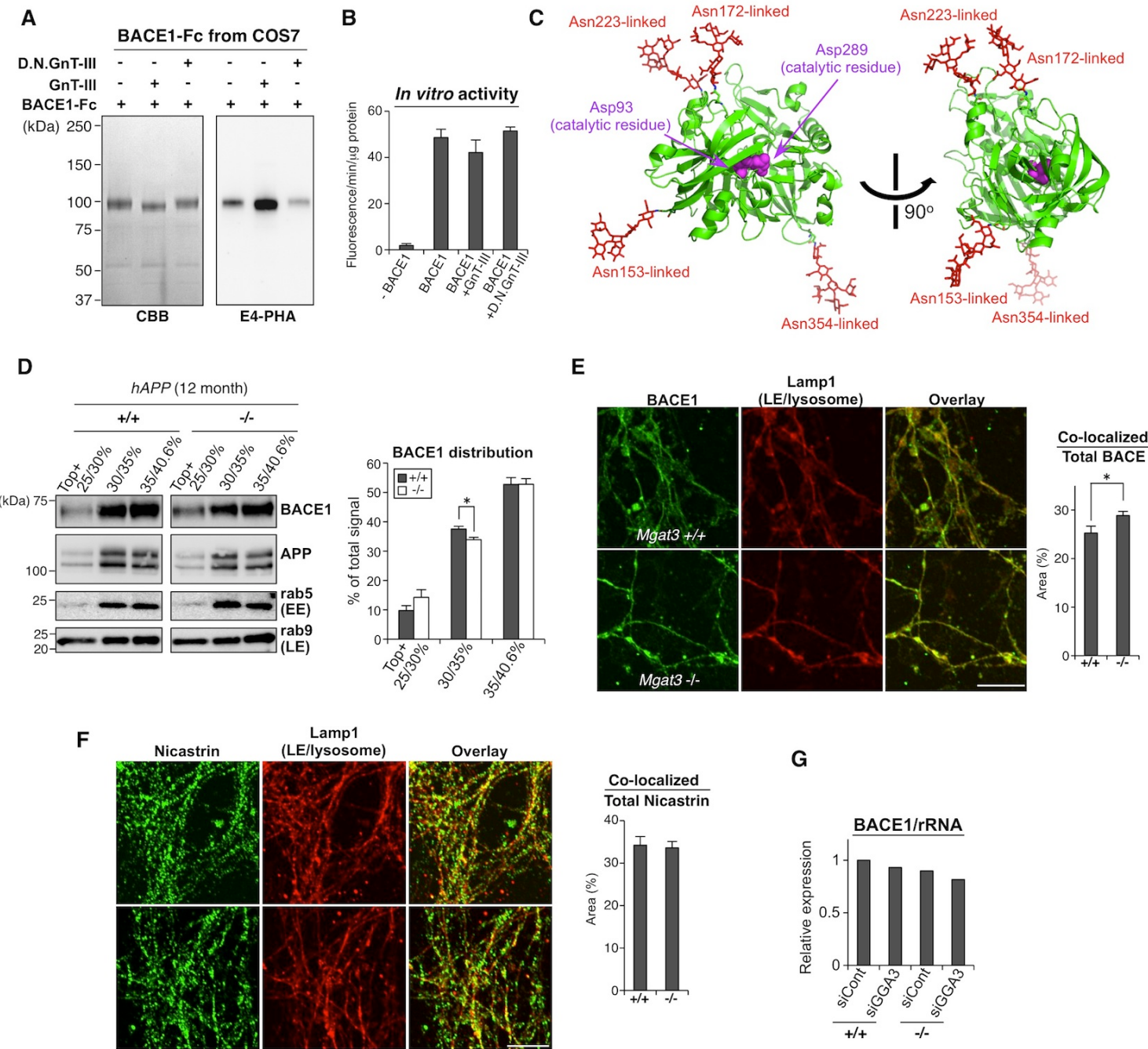

Supplement: Supplementary file 1 [file emmm0007-0175-sd1.pdf]

**Fig.S1A**

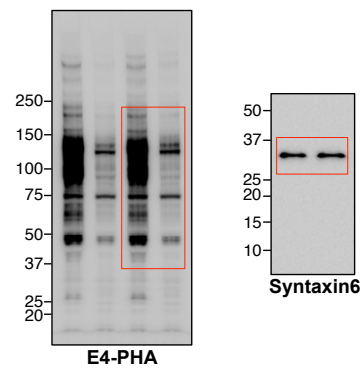

**Fig.S1D**

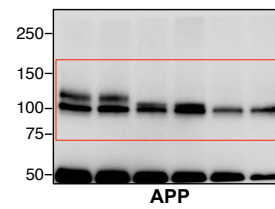

**Fig.S1E**

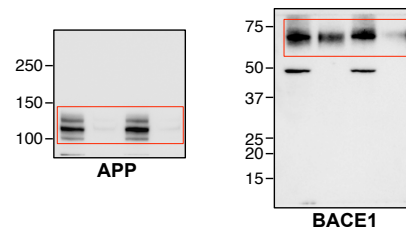

**Fig.S1F**

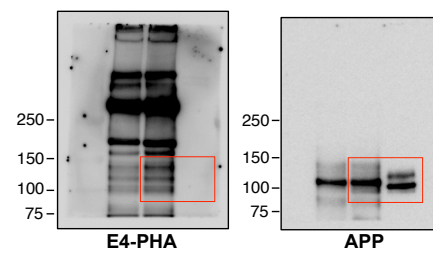

**Fig.S1G**

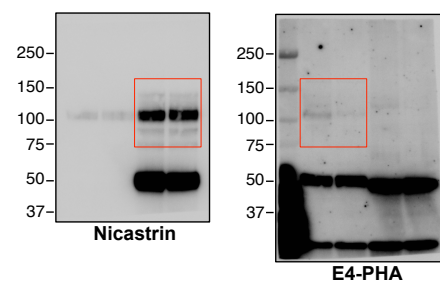

Supplement: Supplementary file 2 [file emmm0007-0175-sd2.pdf]

**Fig.1B**

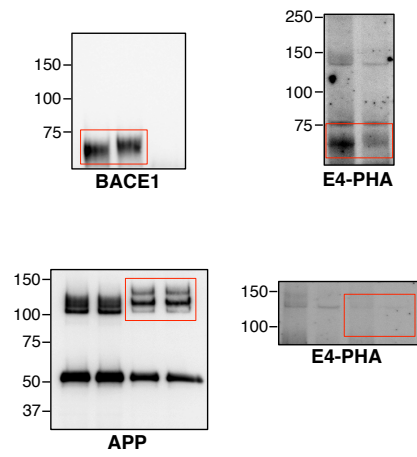

**Fig.1C**

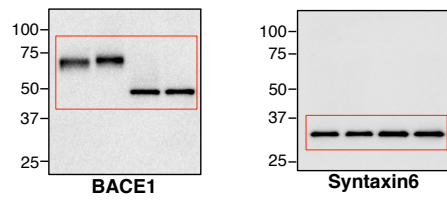

Supplement: Supplementary file 5 [file emmm0007-0175-sd5.pdf]

**Fig.2B**

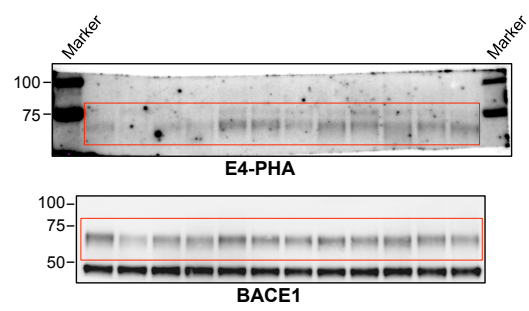

Supplement: Supplementary file 6 [file emmm0007-0175-sd6.pdf]

**Fig.3A**

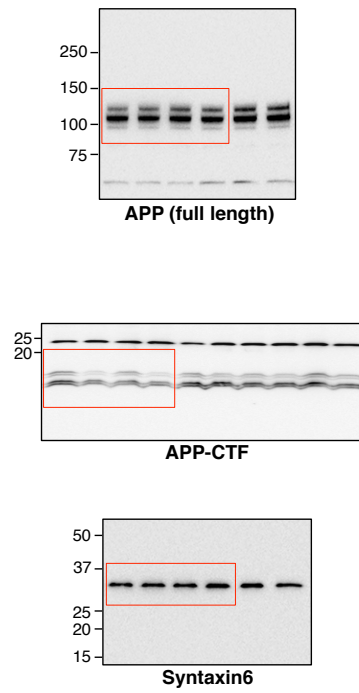

**Fig.3B**

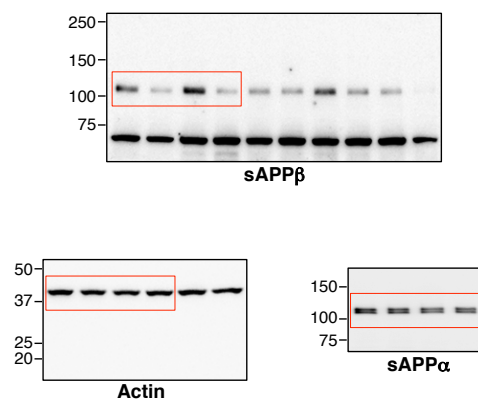

Supplement: Supplementary file 7 [file emmm0007-0175-sd7.pdf]

Fig.5A

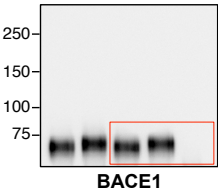

Fig.5B

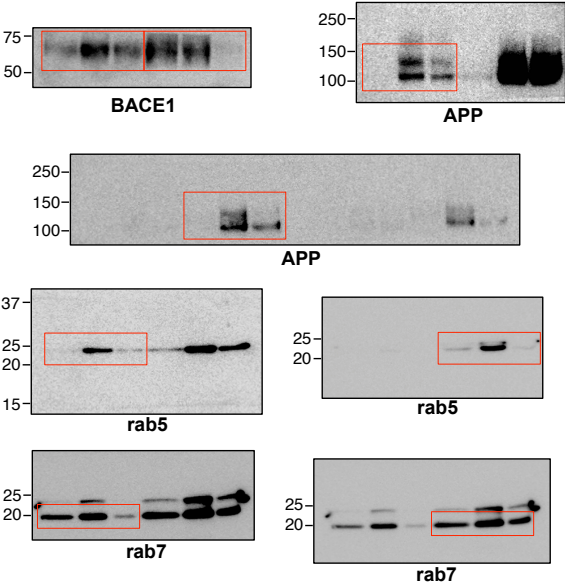

Fig.5C

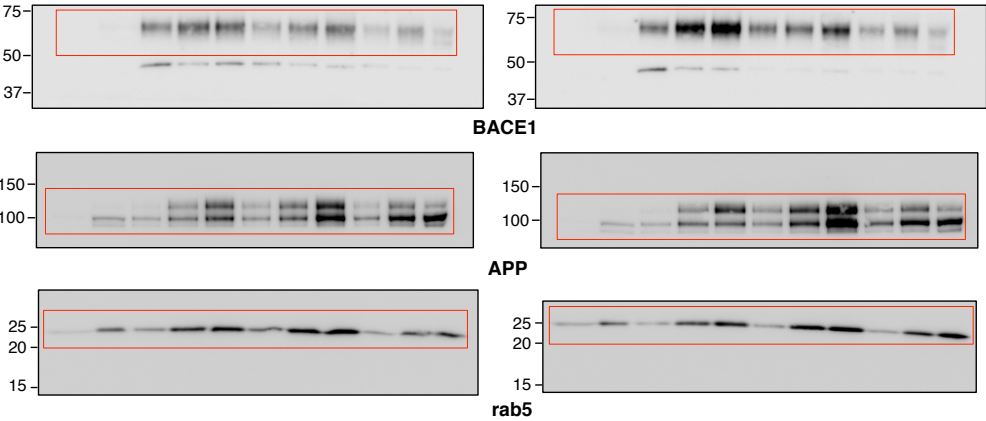

Fig.5E

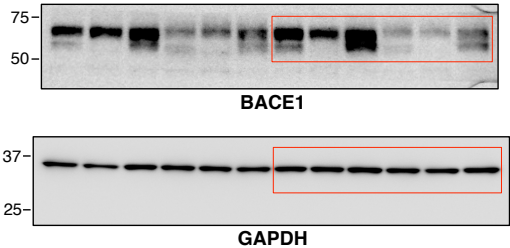

Supplement: Supplementary file 8 [file emmm0007-0175-sd8.pdf]

**Fig.6B**

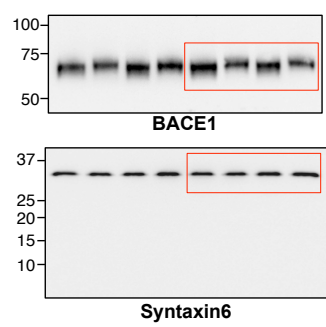

**Fig.6C**

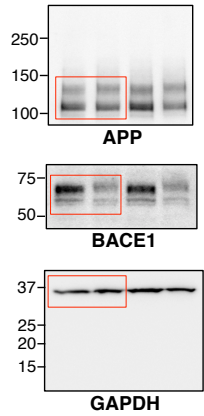

**Fig.6E**

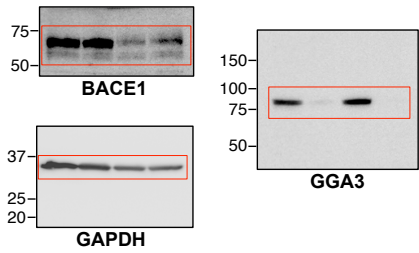

Supplement: Supplementary file 9 [file emmm0007-0175-sd9.pdf]

**Fig.7A**

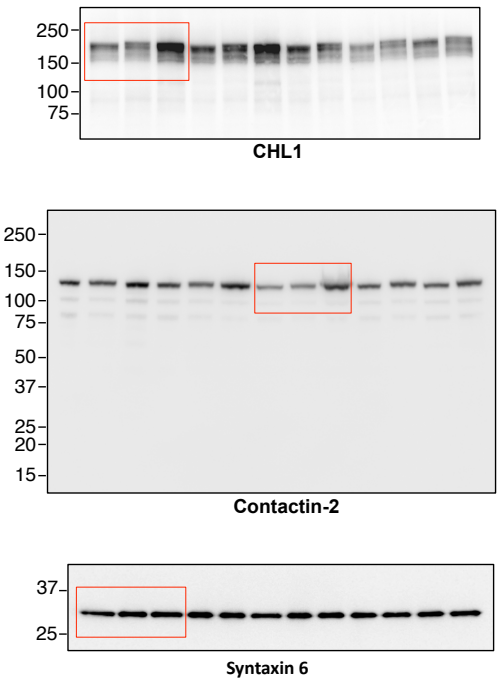

Supplement: Supplementary file 10 [file emmm0007-0175-sd10.pdf]
