# Supplementary material for: An aberrant sugar modification of BACE1 blocks its lysosomal targeting in Alzheimer's disease
Source: EMBO Mol Med. 2015 Jan 15;7(2):175–89. doi: 10.15252/emmm.201404438 (PMC4328647; doi:10.15252/emmm.201404438)
Supplement: Supplementary file 3 [file emmm0007-0175-sd3.docx]

**Supplementary Figure legends**

**Supplementary Figure S1. E4-PHA lectin recognizes BACE1 in neurons.** (A) Proteins from a brain membrane fraction of 5-month-old *hAPP/Mgat3^+/+^* or *hAPP/Mgat3^-/-^* mice were blotted with E4-PHA lectin or syntaxin 6 (loading control). Asterisks indicate non-specific bands, which reacted with HRP-avidin. (B) Staining of 12-month-old *hAPP/Mgat3^+/+^* or *hAPP/Mgat3^-/-^* mouse brain sections (hippocampus or cerebral cortex) with E4-PHA lectin. Scale bar, 100 μm. (C) Double-staining of 12-month-old *hAPP/Mgat3^+/+^* or *hAPP/Mgat3^-/-^* mouse brain sections with E4-PHA lectin and anti-cell marker antibodies (microtubule-associated protein 2 (MAP2) for neurons, glial fibrillary acidic protein (GFAP) for astrocytes, myelin basic protein (MBP) for oligodendrocytes or ionized calcium binding adaptor molecule 1 (Iba1) for microglia). Scale bar, 100 μm. (D) Immunoprecipitated APP was treated with sialidase with or without *O*-glycosidase, and then immunoblotted. (E) Proteins from brain membrane fractions of 3-month-old *hAPP/Mgat3^+/+^* or *hAPP/Mgat3^-/-^* mice were extracted (input) and then incubated with E4-PHA agarose. Bound proteins were eluted and analyzed by western blotting with anti-BACE1 or anti-APP C-term antibody (F) APP was immunoprecipitated from C17 cells or 19-week-old *hAPP/Mgat3^+/+^* mouse brains and blotted with E4-PHA lectin (lower) or anti-APP antibody (upper). (G) Nicastrin was immunoprecipitated from 3-month-old *hAPP/Mgat3^+/+^* or *hAPP/Mgat3^-/-^*mouse brains and blotted with E4-PHA lectin (lower) or anti-nicastrin antibody (upper).

**Supplementary Figure S2. BACE1 is modified with bisecting GlcNAc in brain.** (A) BACE1 was immunoaffinity purified from 1-week-old mouse brains. Purity was demonstrated by silver staining. The procedure for subsequent glycan analyses of BACE1 and anti-BACE1 IgG is also illustrated. Anti-BACE1 IgG was analyzed to rigorously exclude the possibility that the identified glycan structures on BACE1 were derived from contaminated anti-BACE1 rabbit IgG used for purification. *N*-glycans were chemically desialylated before LC-MS analysis. (B,C) Average MS spectrum of desialo-alditol *N*-glycans from mouse brain BACE1 (B) or anti-BACE1 rabbit IgG heavy chain (C). Asterisks indicate peaks with an unknown 31 Da adduct. (D-H) MS/MS spectrum of *m/z* 913.838 ion (D), *m/z* 986.868 ion (E), *m/z* 1015.380 ion (F), *m/z* 1096.404 ion (G), or *m/z* 1250.460 ion (H). Diagnostic ions showing the presence of bisecting GlcNAc were observed.

**Supplementary Figure S3. Synaptic loss and astroglial accumulation around Aβ plaques in *hAPP/Mgat3^+/+^* mouse brain** (A) Double-staining of 12-month-old *hAPP/Mgat3^+/+^* or *hAPP/Mgat3^-/-^* mouse brain sections (cerebral cortex) for Aβ plaque and the postsynaptic marker PSD95. Scale bar, 50 μm. (B, C) Double-staining of 12-month-old *hAPP/Mgat3^+/+^* or *hAPP/Mgat3^-/-^* mouse brain sections (cerebral cortex) for Aβ plaque and the astroglial marker GFAP. Scale bar, 100 μm. Arrows indicate the location of Aβ plaque. Scale bar, 100 μm. The GFAP signal intensity was quantified (*n* = 4). The graph on the right shows means ± SEM (**p* < 0.05, Student’s *t*-test, *p* = 0.021).

**Supplementary Figure S4. Bisecting GlcNAc regulates BACE1 distribution but not its catalytic activity.** (A) Soluble BACE1-Fc was co-expressed with GnT-III or dominant negative (D.N.) GnT-III in COS-7 cells and purified from culture medium through a protein G column. The purity of BACE1-Fc and its reactivity with E4-PHA were confirmed by Coomassie Brilliant Blue staining (left) and E4-PHA blotting (right). (B) The activity of BACE1-Fc, measured using fluorogenic APP peptide, is shown as means ± SEM (*n* = 3). (C) Three-dimensional structural model of human BACE1 incorporating bisected *N*-glycans. The polypeptide is shown in green (ribbon model) and the bisected *N*-glycans are shown in red (stick representation). The catalytic residues, Asp93 and Asp289, are shown in magenta (space-filling model). The figure was prepared with the PyMOL Molecular Graphics System, Version 0.99. (D) Brain homogenates were fractionated by sucrose density centrifugation and immunoblotted for BACE1, APP, rab5 or rab9. EE, early endosome. LE, late endosome. Signal intensity of BACE1 in each fraction was quantified and shown as the graph (*n* = 4). (E) BACE1 was expressed in primary neurons by lentiviral infection, and then co-immunostained with Lamp1. The area in which co-localized staining was observed was quantified as a percentage of the total BACE1-positive area (right, *n* = 10). Scale bar, 10 μm. (F) Immunostaining of primary neurons for nicastrin and Lamp1. Scale bar, 10 μm. The area in which co-localized staining was observed was quantified as a percentage of the total nicastrin-positive area (right, *n* = 8). (G) BACE1 mRNA levels relative to rRNA were measured in MEF cells treated with control siRNA or GGA3-targeting siRNA. BACE1 expression was not increased by GGA3 knockdown in either case (*n* = 2). All graphs show means ± SEM (**p* < 0.05, Student’s *t*-test, *p* = 0.093 for Top+25/30%, *p* = 0.014 for 30/35%, *p* = 0.319 for 35/40% in (D), *p* = 0.045 for (E)).
