# Supplementary material for: An aberrant sugar modification of BACE1 blocks its lysosomal targeting in Alzheimer's disease
Source: EMBO Mol Med. 2015 Jan 15;7(2):175–89. doi: 10.15252/emmm.201404438 (PMC4328647; doi:10.15252/emmm.201404438)
Supplement: Supplementary file 4 [file emmm0007-0175-sd4.pdf]

An aberrant sugar modification of BACE1 blocks its lysosomal targeting in Alzheimerís disease

Yasuhiko Kizuka, Shinobu Kitazume, Reiko Fujinawa, Takashi Saito, Nobuhisa Iwata, Takaomi C. Saido, Miyako Nakano, Yoshiki Yamaguchi, Yasuhiro Hashimoto, Matthias Staufenbiel, Hiroyuki Hatsuta, Shigeo Murayama, Hiroshi Manya, Tamao Endo, Naoyuki Taniguchi

Corresponding author: Naoyuki Taniguchi, RIKEN Global Research Cluster

Review timeline:

Submission date:	04 August 2014
Editorial Decision:	28 August 2014
Revision received:	30 October 2014
Editorial Decision:	21 November 2014
Revision received:	02 December 2014
Accepted:	12 December 2014

Transaction Report:

(Note: With the exception of the correction of typographical or spelling errors that could be a source of ambiguity, letters and reports are not edited. The original formatting of letters and referee reports may not be reflected in this compilation.)

Editor: Roberto Buccione

1st Editorial Decision 28 August 2014

Thank you for the submission of your manuscript to EMBO Molecular Medicine.

In this case we experienced unusual difficulties in securing three willing and appropriate reviewers. As a further delay cannot be justified I have decided to proceed based on the two available consistent evaluations.

Both Reviewers find merits in your manuscript although they raise significant issues that require your action. I will not dwell into much detail as their comments are detailed. I would like, however, to highlight a few main points.

Reviewer 1, as you will see, has two orders of concerns. On one hand s/he notes that the findings described in this manuscript are in stark contrast with previous work and suggests a number of avenues to directly address this issue. On the other hand, the Reviewer notes inadequate experimental support for some of the claims. One important example is that the Mgat3 GOF/LOF experiments should be performed in neurons. I should mention here that Reviewer 2 has exactly the same concern. Another important caveat, and I agree, is that to correctly assess the clinical relevance of your findings, it would be important to assess the consequences of BACE1 modification also on substrates other than APP. Finally, this Reviewer also mentions that the effects of Mgat3 deficiency should be assessed on an alternative mouse setting to ascertain whether the effects are model specific. This Reviewer lists many other action points for your consideration.

Reviewer 2, as mentioned above and similarly to Reviewer 1, notes that the claims should be verified in a neuronal setting. S/he is also concerned, among other things, that the experimental evidence does not support a model based on BACE1 levels but rather on its localization: this aspect should be convincingly clarified. This Reviewer also notes the non-standard approach to Abeta analysis and would like to see this resolved.

In conclusion, while publication of the paper cannot be considered at this stage, we would be prepared to consider a substantially revised submission, with the understanding that the Reviewers' concerns must be fully addressed with additional experimental data where appropriate and that acceptance of the manuscript will entail a second round of review.

The required revision in this case appears to require a significant amount of time, additional work and experimentation and might be technically challenging. I would therefore understand if you chose to rather seek publication elsewhere at this stage. Should you do so, we would welcome a message to this effect.

Please note that it is EMBO Molecular Medicine policy to allow a single round of revision only and that, therefore, acceptance or rejection of the manuscript will depend on the completeness of your responses included in the next, final version of the manuscript.

As you know, EMBO Molecular Medicine has a "scooping protection" policy, whereby similar findings that are published by others during review or revision are not a criterion for rejection. However, I do ask you to get in touch with us after three months if you have not completed your revision, to update us on the status. Please also contact us as soon as possible if similar work is published elsewhere.

We look forward to reading your revised manuscript in due time.

***** Reviewer's comments *****

Referee #1 (Remarks):

Kizuka and colleagues provide an exciting new mechanism controlling the activity of the beta-secretase BACE1, which is a key drug target in Alzheimer's disease (AD). The authors demonstrate that BACE1 carries a particular sugar modification as a result of an enzyme called Mgat3. This modification slows lysosomal degradation of BACE1, resulting in i) slightly altered cellular localization of BACE1, ii) increased BACE1 protein levels (at least in older mice) and iii) higher levels of the BACE1 cleavage product Abeta, which is the pathogenic peptide in AD. The authors report the modification to be increased in AD and find that mice deficient in Mgat3 have strongly reduced amyloid pathology, neuroinflammation and cognitive dysfunction. This implies that Mgat 3 may potentially be involved in AD. More importantly, the findings clearly demonstrate that Mgat3 may well serve as a new drug target in AD. Thus, this novel and timely work provides a solid basis for further mechanistic and preclinical studies on Mgat3 in AD. The manuscript is well written, but I cannot judge the quality of the glycan mass spec data, with which I am not familiar.
My major concern, however, is two-fold. First, two major findings of the manuscript are opposite to the previous findings by one of the authors on the current manuscript (Akasaka-Manya et al. Glycobiology 2010). While the older manuscript reported that Mgat3 expression needs to be enhanced in order to reduce ABeta levels, the current manuscript reports the opposite. Likewise the older manuscript reported that APP is modified by bisecting GlcNAc, the current manuscript claims that this is not the case. These opposing findings are not even discussed. Second, several controls are missing and some of the conclusions are overstated or not yet justified by the data.

The following major and minor points need to be addressed:

1. To resolve the discrepancies with the previous manuscript the authors should repeat the analysis

of APP glycosylation (bisecting GlcNAc yes or no?) in the cell line used in the previous study. That analysis will reveal whether this APP modification is cell-type specific (neurons versus peripheral cells), which may even shed further light (besides BACE1 expression levels) on why Abeta production is high in the brain but low in the periphery. The authors argue that the lack of mobility shift of APP is evidence for APP not being modified. However, it is well known that the apparent molecular weight of APP is primarily determined by O- and not by N-glycosylation. Thus, if the authors want to confirm this claim, they need to remove the O-glycosylation and may then be able to detect the mobility shift.
2. To clarify the other central discrepancy to the previous manuscript, the authors need to clarify whether overexpression of Mgat3 increases or decreases (or does not alter) ABeta levels and other APP parameters. IN parallel modification of BACE1 and APP by bisecting GlcNAc need to be measured. This will a) clarify the discrepancy with the previous study and b) potentially allow a new interpretation of the mechanism of bisecting GlcNAc on APP processing. If both overexpression and knock-down of Mgat3 reduce Abeta levels, the modification itself cannot be simply bad or good, but instead the right amount of the modification is required. This, however, would question the proposed mechanism of Mgat3 on APP processing. Overexpression should be done in primary neurons in order to be able to compare the new data to the current manuscript.
3. The authors use one AD mouse model. Given that AD mouse models differ significantly from each other and given the discrepancies between the current and the previous study, the authors need to analyze the effect of Mgat3-deficiency in wild-type mice or in another AD mouse model. This will rule out the possibility that the observed effects are only true in the model used here. From Figure 6 it seems that endogenous BACE1 and Abeta can be measured from MEF cells. Thus, the easiest approach would be to measure both parameters also in brain extracts from wild-type mice.
4. The authors claim that the effect of the BACE1 modification is selective on the BACE1 substrate APP and does not act on other BACE1 substrates. This is a very bold statement given the wealth of other BACE1 substrates. In particular CHL1 may not be the best substrate for this kind of analysis as a lack of CHL1 cleavage by BACE1 can be compensated for by other proteases. The authors should test at least another BACE1 substrate, such as L1 or the sialyltransferase which they found to be a BACE1 substrate many years ago.
5. The in vitro assay in Figure 5B is not convincing. Even in the absence of BACE1 the authors still measure about 50% of BACE1 activity (compared to the controls). This clearly shows that the assay is not specific for BACE1, but probably also measures cathepsin D activity. The value for the BACE1 knock-out appears to be the background value. Since this is the maximum reduction possible in the assay, the statistical data need to be correlated to this maximally possible reduction, which may yield a statistically significant reduction in activity for the Mgat3-/- compared to the control. Alternatively, the assay could be performed with the substrate (fusion) protein and not just with a synthetic peptide, which should help to discriminate between true BACE1 and cathepsin B cleavages.
6. Immunofluorescence pictures are difficult to quantify and the changes in Figure 5D and E4B are difficult to judge. The authors need to stain for another membrane protein and demonstrate that the mild effects are indeed seen for BACE1 but not for any other membrane protein.

Minor points:

1. The authors provide a structural model in which the sugars are distant from the active site of BACE1. While this fits with the solved BACE1 crystal structure (where the soluble BACE1 ectodomain was used), it is taken as evidence that the sugars (and thus the new modification) should not alter BACE1 activity. However, full-length transmembrane BACE1 is active as a dimer and not as a monomer, as shown by the Haass and Multhaup labs. The dimer has a higher activity than the monomer. Thus, it remains possible that dimerization and thus activity are affected by the sugars. This sentence in the second last paragraph on page 8 should be toned down.
2. Citation of the old Vassar review article on BACE1 should be replaced by one of the more recent Vassar review articles (2013 or 2014) or newer articles from other colleagues.
3. On page 10 the three recent articles on CHL1 identification as a BACE1 substrate should be cited (Hitt et al. JBC2013; Kuhn et al. EMBO 2012, Zhou et al. JBC 2012).
4. The authors claim that Mgat3-deficient mice do not show a severe phenotype and try to make a strong case for Mgat3 inhibition being superior to BACE1 inhibition. However, a previous publication from the Stanley lab (2002) reported mild neurological deficits at least in Mgat3-mutant mice. This should be discussed more carefully and be contrasted with the phenotype of BACE1-deficient mice.

5. In Figure E3B it is obvious that there is not GFAP staining in areas where there are no plaques (in the Mgact3-deficient mice). However, plaques are still seen (albeit reduced) in the Mgat3-/- mice. The authors should include a panel where GFAP staining is shown in the vicinity of one of the remaining plaques. Quantification is then needed to demonstrate that indeed GFAP staining in total is much less in the -/- mice.

Referee #2 (Comments on Novelty/Model System):

MS analysis of glycation of BACE1 is modern, while the authors seem unfamilier with the standard analysis of APP-transgenic mÌce. The glycation of AD-associated molecules are less investigated, although this topic is very interesting. This study is aimed to find a target specifically inhibiting Abeta generation without severe side-effects. The authors have used knockout mouse and cell models. However, they misused fibroblasts instead of neurons or neuronal tissues for BACE1 analysis.

Referee #2 (Remarks):

Kizuka et al. have observed that: Sugar modification of BACE1 is increased in AD; This modification affects BACE1 localization in endosomes and its lysosomal degradation; The different location of BACE1 and APP leads to less Abeta production; This sugar modification specifically affects the interaction between BACE1 and APP. The topic is very interesting. However, the study appears quite preliminary.
1) The authors have observed that GlcNAc-transferase is up-regulated in AD patients. BACE1 is modified by this enzyme. How about other enzymes? Especially gamma secretase complex? It should be included as a control.
2) The demonstration of BACE1 localization is not sufficient. Fig.5B is convincing, however, in Fig. 5D, it is very difficult to conclude that the colocalization of BACE1 and LAMP1 in Mgat3-deficient and wildtype brains is different. Other convincing methods are needed.
3) The results regarding Abeta generation, BACE1 activity and BACE1 degradation are not on the same line. Fig.1B shows BACE1 modification, Fig. 3 shows BACE1 activity and Fig.4 shows Abeta concentration in the brain. All these data is derived from 3-month-old APP mice. However, the authors described that there is no reduction of BACE1 proteins in 3-month-old APP-transgenic mice, which suggests that the altered Abeta generation is due to the different location of APP and BACE1. Thus, the title of this manuscript should be changed. The cortex and hippocampus should be used to investigate the location of APP and BACE1 instead of immortalized fibroblasts (Fig.5B).
4) The protein level of BACE1 presented in Fig.5C could be equal between Mgat3-deficient and wildtype fibroblasts, which is controversial to Fig.6C. Because of no clear correlation between BACE1 modification and degradation, the reviewer wonders whether the hypothesis on BACE1 location in the late endosome is correct.
5) The Abeta assay should be performed according to the standard protocols. Guanidine-soluble fraction does not reflect the membrane-associated Abeta. The results between the concentrations of Abeta in TBS-fraction and Guanidine-fraction might be larger. In Fig. 4C, no plaques in hAPP/Magt3-/- mice could be seen. It is very difficult to believe that the Abeta generation is nearly completely blocked when the sugar modification on BACE1 is ablated, whereas, in Fig. 5B, there are still sufficient BACE1 and APP located in the early endosome. It could be better for authors to present the location of APP and BACE1 in 12-month-old APP-transgenic mouse brain.
6) The description of synaptic impairment and astrocytes is wrong in Fig. E3. Please use the published protocols.
7) The abbreviation, for example, E4-PHA on page 6, should be expanded when it was first used.

1st Revision - authors' response 30 October 2014

Referee #1 (Remarks):

Kizuka and colleagues provide an exciting new mechanism controlling the activity of the beta-secretase BACE1, which is a key drug target in Alzheimer's disease (AD). The authors demonstrate that BACE1 carries a particular sugar modification as a result of an enzyme called Mgat3. This modification slows lysosomal degradation of BACE1, resulting in i) slightly altered cellular localization of BACE1, ii) increased BACE1 protein levels (at least in older mice) and iii) higher levels of the BACE1 cleavage product Abeta, which is the pathogenic peptide in AD. The authors report the modification to be increased in AD and find that mice deficient in Mgat3 have strongly reduced amyloid pathology, neuroinflammation and cognitive dysfunction. This implies that Mgat3 may potentially be involved in AD. More importantly, the findings clearly demonstrate that Mgat3 may well serve as a new drug target in AD. Thus, this novel and timely work provides a solid basis for further mechanistic and preclinical studies on Mgat3 in AD. The manuscript is well written, but I cannot judge the quality of the glycan mass spec data, with which I am not familiar.
My major concern, however, is two-fold. First, two major findings of the manuscript are opposite to the previous findings by one of the authors on the current manuscript (Akasaka-Manya et al. Glycobiology 2010). While the older manuscript reported that Mgat3 expression needs to be enhanced in order to reduce ABeta levels, the current manuscript reports the opposite. Likewise the older manuscript reported that APP is modified by bisecting GlcNAc, the current manuscript claims that this is not the case. These opposing findings are not even discussed. Second, several controls are missing and some of the conclusions are overstated or not yet justified by the data.

The following major and minor points need to be addressed:

Comment 1.
To resolve the discrepancies with the previous manuscript the authors should repeat the analysis of APP glycosylation (bisecting GlcNAc yes or no?) in the cell line used in the previous study. That analysis will reveal whether this APP modification is cell-type specific (neurons versus peripheral cells), which may even shed further light (besides BACE1 expression levels) on why Abeta production is high in the brain but low in the periphery. The authors argue that the lack of mobility shift of APP is evidence for APP not being modified. However, it is well known that the apparent molecular weight of APP is primarily determined by O- and not by N-glycosylation. Thus, if the authors want to confirm this claim, they need to remove the O-glycosylation and may then be able to detect the mobility shift.

Response:
In order to address whether or not APP glycosylation is cell-type specific, we have analyzed the level of bisecting GlcNAc on APP in C17 cells (the cell line used in the previous paper, Akasaka-Manya et al., *Glycoconj. J.*, 2008, 775-786) and in brain. APP immunoprecipitated from each sample was stained with E4-PHA lectin. Although we detected equivalent levels of APP, we did confirm that brain APP was not detected with E4-PHA whereas a clear E4-PHA signal was detected for APP in C17 cells (Expanded Fig. E1F) (page 6 lines 13-16). This analysis therefore revealed that APP glycosylation is indeed cell-type specific, a point which we now discuss on page 12 lines 8-12. Furthermore, even after APP O-glycans were removed by treatment with sialidase and O-glycosidase, mobility shift of APP was not observed in *Mgat3*$^{-/-}$ brains (Expanded Fig. E1D) (page 6 lines 9-10). These results support our conclusion that APP is not modified or is barely modified with bisecting GlcNAc in the brain.

Comment 2.
To clarify the other central discrepancy to the previous manuscript, the authors need to clarify whether overexpression of Mgat3 increases or decreases (or does not alter) Abeta levels and other APP parameters. In parallel, modification of BACE1 and APP by bisecting GlcNAc need to be measured. This will a) clarify the discrepancy with the previous study and b) potentially allow a new interpretation of the mechanism of bisecting GlcNAc on APP processing. If both overexpression and knock-down of Mgat3 reduce Abeta levels, the modification itself cannot be simply bad or good, but instead the right amount of the modification is required. This, however, would question the proposed mechanism of Mgat3 on APP processing. Overexpression should be done in primary neurons in order to be able to compare the new data to the current manuscript.

Response:
In order to address the reviewer's concern, we overexpressed GnT-III in mouse primary neurons and found a reduction in Ab, as previously reported (Akasaka-Manya et al., *Glycobiology*, 2010, 99-106). (Fig. R1A). Moreover, we found that *Mgat3*$^{-/-}$ neurons produced lower levels of Ab than *Mgat3*$^{+/+}$ neurons, although the rate of reduction was less than in mouse brain samples (Fig. R1B). These results actually fit the reviewer's comment that both overexpression and knockdown of Mgat3 could reduce Ab levels. What we would like to point out, however, is that an extremely high level of GnT-III is expressed in these overexpression experiments. Our previous study reported that GnT-III overexpression led to an ~320 fold increase in GnT-III enzyme activity, whereas the level of GnT-III mRNA in AD brains is only ~1.5 times higher than that in control brains. We have found that such GnT-III overexpression largely impairs BACE1 enzyme activity in vitro (Fig. R2), suggesting that excessive BACE1 modification with bisecting GlcNAc could affect the conformation of BACE1, impairing its catalytic activity. The reason why the reduction of BACE1-Fc enzyme activity by GnT-III overexpression was lower (Expanded Fig. E4A, B) is probably because only properly folded BACE1-Fc is secreted in this situation. When GnT-III was overexpressed in COS cells, we found that the level of BACE1-Fc in the medium was less than half of that in control cells, supporting our speculation that GnT-III overexpression at extremely high level interferes with the folding of BACE1.

Fig. R1

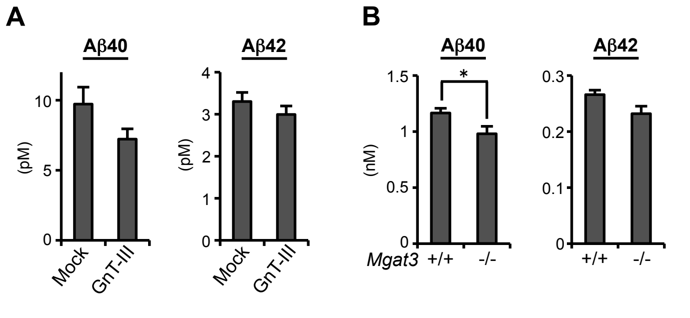

(A) Neurons (DIV4) were transfected with the plasmid encoding human GnT-III. After 3 days, the concentration of Aβ in the culture medium was measured (*n* = 3). (B) The Aβ concentration in the medium of primary neuronal cultures (DIV11) was also measured (*n* = 3, $^{*}p < 0.05$, Student's *t*-test).

Unfortunately, we found that the levels of BACE1 and APP in neurons were too low to detect bisecting GlcNAc modification, partly because a transfection reagent caused neuronal damage. Therefore, to check the glycosylation states of these proteins in GnT-III-overexpressing cells, we used Neuro2A cells as, similar to neurons, the level of bisecting GlcNAc on APP is negligible in these cells (Fig. R3A). Consistent with the impaired BACE1 activity (Fig. R2), overexpression of GnT-III significantly reduced both Ab and sAPPb in Neuro2A cells (Fig. R3B, C). As expected, GnT-III overexpression increased the level of bisecting GlcNAc on BACE1, but APP still reacted poorly with E4-PHA (Fig. R3A). This result indicates that APP in Neuro2A cells is hardly modified by overexpressed GnT-III, again confirming our finding that glycan modification by GnT-III is cell type- and protein-specific, although the mechanism for this interesting phenomenon remains unclear at present.

Fig. R2

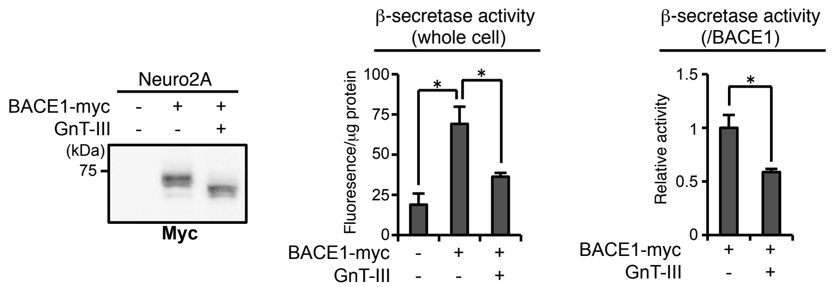

Neuro2A cells were transfected with BACE1-myc and/or GnT-III plasmids, after which cell lysates were blotted with anti-myc antibody (left). Specific activity of β-secretase in the cell lysates was measured (*n* = 3) (middle). The activity of the mock samples were subtracted as the background, after which the resultant value was normalized by the level of expressed BACE1 (signal intensity in myc western blot) to directly compare the activity of expressed BACE1 (right) (*n* = 3, $^{*}p < 0.05$, Student's *t*-test).

Fig. R3

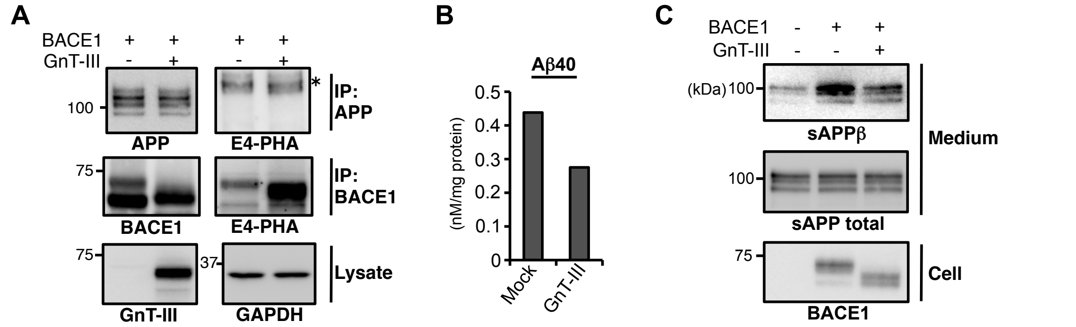

(A) hBACE1 was overexpressed with or without hGnT-III in Neuro2A cells. Cell lysates were western blotted for GnT-III and GAPDH (lower). APP or BACE1 was immunoprecipitated, and then blotted with E4-PHA lectin (upper and middle). An asterisk indicates a non-specific band. (B) Neuro2A cells were transfected with GnT-III plasmid, after which the concentration of Aβ40 in the culture medium was measured. (C) hBACE1 was overexpressed with or without hGnT-III in Neuro2A cells. Proteins secreted into the culture medium were western blotted for sAPPβ or total sAPP. The cell lysates were also immunoblotted for BACE1.

Comment 3.
The authors use one AD mouse model. Given that AD mouse models differ significantly from each other and given the discrepancies between the current and the previous study, the authors need to analyze the effect of Mgat3-deficiency in wild-type mice or in another AD mouse model. This will rule out the possibility that the observed effects are only true in the model used here. From Figure 6 it seems that endogenous BACE1 and Abeta can be measured from MEF cells. Thus, the easiest approach would be to measure both parameters also in brain extracts from wild-type mice.

Response:
We have now measured Ab levels in non-transgenic *Mgat3*$^{+/+}$ and *Mgat3*$^{-/-}$ brains as suggested, and found that Ab is slightly but significantly reduced in non-APP transgenic *Mgat3*$^{-/-}$ mice compared with *Mgat3*$^{+/+}$ mice (Fig. 4C) (page 8 lines 10-13), supporting our conclusion. Combined with the fact that Ab reduction in aged *hAPP/Mgat3*$^{-/-}$ mice is more drastic than in young mice (Fig. 4A, B), another factor (e.g. oxidative stress) probably exists which promotes the pathological effect of bisecting GlcNAc on BACE1 in *hAPP/Mgat3*$^{+/+}$ mice. We intend to investigate the mechanisms underlying these phenomena in a future study.

Comment 4.
The authors claim that the effect of the BACE1 modification is selective on the BACE1 substrate APP and does not act on other BACE1 substrates. This is a very bold statement given the wealth of other BACE1 substrates. In particular CHL1 may not be the best substrate for this kind of analysis as a lack of CHL1 cleavage by BACE1 can be compensated for by other proteases. The authors should test at least another BACE1 substrate, such as L1 or the sialyltransferase which they found to be a BACE1 substrate many years ago.

Response:
As the reviewer pointed out, selectivity of BACE1 dysfunction to APP might have been overstated because BACE1 has many physiological substrates other than CHL1. We have toned down our statement by adding "somewhat" in the results (page 11 line 8). In addition, we analyzed the cleavage of another BACE1 substrate, contactin-2, confirming that, similar to CHL1, it is almost normally cleaved in *Mgat3*$^{-/-}$ brain (Fig. 7A) (page 11 line 14).

Comment 5.
The in vitro assay in Figure 5B is not convincing. Even in the absence of BACE1 the authors still measure about 50% of BACE1 activity (compared to the controls). This clearly shows that the assay is not specific for BACE1, but probably also measures cathepsin D activity. The value for the BACE1 knock-out appears to be the background value. Since this is the maximum reduction possible in the assay, the statistical data need to be correlated to this maximally possible reduction, which may yield a statistically significant reduction in activity for the Mgat3-/- compared to the control.

Alternatively, the assay could be performed with the substrate (fusion) protein and not just with a synthetic peptide, which should help to discriminate between true BACE1 and cathepsin B cleavages.

Response:
We have changed the graph by setting the value of the BACE1 knockout sample as the background, and compared these values between *Mgat3*$^{+/+}$ and *Mgat3*$^{-/-}$ samples. However, the difference still did not reach statistical significance (Fig. 5A).

Comment 6. Immunofluorescence pictures are difficult to quantify and the changes in Figure 5D and E4B are difficult to judge. The authors need to stain for another membrane protein and demonstrate that the mild effects are indeed seen for BACE1 but not for any other membrane protein.

Response:
As a control membrane glycoprotein, nicastrin was immunostained, and we confirmed that co-localization of nicastrin and Lamp1 is not changed between *Mgat3*$^{+/+}$ and *Mgat3*$^{-/-}$ brains and neurons (Fig. 5G and Expanded Fig. E4F) (page 10 lines 8-12), supporting our conclusion that more BACE1 is localized to late endosomes / lysosomes in *Mgat3*$^{-/-}$ cells. Moreover, as suggested by reviewer 2, we analyzed BACE1 distribution using another approach (sucrose density fractionation) to strengthen our conclusion regarding the altered localization of BACE1 in the brain. Consistent with the immunofluorescence data and MEF results, the fractionation experiments also showed that BACE1 distribution is altered in *hAPP/Mgat3*$^{-/-}$ brains in both 3-month and 12-month-old mice (Fig. 5C and Expanded Fig. E4D) (page 9 lines 1-3 from the bottom). In addition, we double-stained APP and BACE1 in brain, and found that co-localization of these two proteins is decreased in *hAPP/Mgat3*$^{-/-}$ brains (Fig. 5D) (page 10 lines 1-2), although APP distribution is not changed, as demonstrated by our fractionation experiments (Fig. 5C).

Taken together, these data support our conclusion that the Ab reduction in *hAPP/Mgat3*$^{-/-}$ brain is caused by the change in localization of BACE1, leading to its reduced co-localization with APP and increased localization to late endosome / lysosomes.

Minor points:
Comment 7.
The authors provide a structural model in which the sugars are distant from the active site of BACE1. While this fits with the solved BACE1 crystal structure (where the soluble BACE1 ectodomain was used), it is taken as evidence that the sugars (and thus the new modification) should not alter BACE1 activity. However, full-length transmembrane BACE1 is active as a dimer and not as a monomer, as shown by the Haass and Multhaup labs. The dimer has a higher activity than the monomer. Thus, it remains possible that dimerization and thus activity are affected by the sugars. This sentence in the second last paragraph on page 8 should be toned down.

Response:
According to the reviewer's suggestion, we have added the following sentence regarding BACE1 dimerization on page 9 lines 11-14, "Therefore, it is unlikely that the enzymatic activity of BACE1 is directly modulated by bisecting GlcNAc, although it is still possible that glycans exposed at the molecular surface exert an indirect effect due to the impaired dimerization of BACE1 (Schmechel et al, 2004; Westmeyer et al, 2004).".

Comment 8.
Citation of the old Vassar review article on BACE1 should be replaced by one of the more recent Vassar review articles (2013 or 2014) or newer articles from other colleagues.

Response:
We have changed the old review to the new one (2014) as suggested (page 4 line 7, page 11 line 10, page 14 line 1).

Comment 9
On page 10 the three recent articles on CHL1 identification as a BACE1 substrate should be cited (Hitt et al. JBC2013; Kuhn et al. EMBO 2012, Zhou et al. JBC 2012).

Response:

We have added the references as suggested (page 11 lines 9-10).

Comment 10.
The authors claim that Mgat3-deficient mice do not show a severe phenotype and try to make a strong case for Mgat3 inhibition being superior to BACE1 inhibition. However, a previous publication from the Stanley lab (2002) reported mild neurological deficits at least in Mgat3-mutant mice. This should be discussed more carefully and be contrasted with the phenotype of BACE1-deficient mice.

Response:
We have added discussion about the phenotypes of mutant mice expressing truncated GnT-III on page 12 line 22 to page 13 line 3 "Although mutant mice expressing truncated GnT-III have been reported to exhibit several neurological defects such as impaired leg clasp reflex, these abnormalities are considerably milder than those observed in *Bace1*$^{-/-}$mice and are not seen in GnT-III-deficient mice (Bhattacharyya et al, 2002), suggesting that they derive from the presence of truncated GnT-III and not from the loss of full-length GnT-III".

Comment 11.
In Figure E3B it is obvious that there is not GFAP staining in areas where there are no plaques (in the Mgact3-deficient mice). However, plaques are still seen (albeit reduced) in the Mgat3-/- mice. The authors should include a panel where GFAP staining is shown in the vicinity of one of the remaining plaques. Quantification is then needed to demonstrate that indeed GFAP staining in total is much less in the -/- mice.

Response:
We have stained brain sections and added new data to cover a broader region in which Ab plaque is also seen in the *hAPP/Mgat3*$^{-/-}$ mouse (Expanded Fig. E3C). The signal intensity of GFAP has been quantified, confirming that it is significantly lower in *hAPP/Mgat3*$^{-/-}$ mice.

Referee #2 (Comments on Novelty/Model System):

MS analysis of glycation of BACE1 is modern, while the authors seem unfamilier with the standard analysis of APP-transgenic mice. The glycation of AD-associated molecules are less investigated, although this topic is very interesting. This study is aimed to find a target specifically inhibiting Abeta generation without severe side-effects. The authors have used knockout mouse and cell models. However, they misused fibroblasts instead of neurons or neuronal tissues for BACE1 analysis.

Referee #2 (Remarks):

Kizuka et al. have observed that: Sugar modification of BACE1 is increased in AD; This modification affects BACE1 localization in endosomes and its lysosomal degradation; The different location of BACE1 and APP leads to less Abeta production; This sugar modification specifically affects the interaction between BACE1 and APP. The topic is very interesting. However, the study appears quite preliminary.

Comment 1.
The authors have observed that GlcNAc-transferase is up-regulated in AD patients. BACE1 is modified by this enzyme. How about other enzymes? Especially gamma secretase complex? It should be included as a control.

Response:
As the reviewer pointed out, nicastrin, which is the only glycosylated subunit in gamma-secretase, could be modified by bisecting GlcNAc. We found that immunoprecipitated nicastrin from the brains of *hAPP/Mgat3*$^{+/+}$ mice but not of *hAPP/Mgat3*$^{-/-}$ mice reacts weakly with E4-PHA lectin (Expanded Fig. E1G) (page 6 lines 16-19), indicating that nicastrin is slightly modified by bisecting GlcNAc. Considering this weak reactivity with E4-PHA in spite of the presence of a much larger number of N-glycans on nicastrin (16 potential sites) as compared with BACE1 (4 sites), nicastrin is

probably a poorer substrate for GnT-III than BACE1. Moreover, previous reports showed that glycosylation of nicastrin is required neither for binding to presenilins nor for activity of gamma-secretase (Herreman et al., *J. Cell Sci.* 2003, 116, 1127-36. Schedin-Weiss et al., *FEBS J.* 2014, 281, 46-62). The absence of an obvious Notch phenotype in *Mgat3*$^{-/-}$ mice also suggests that gamma-secretase activity is not modulated by the lack of bisecting GlcNAc. Based on these facts, we think that the lack of bisecting GlcNAc modification on nicastrin is not the major reason for the reduced Ab generation in *Mgat3*$^{-/-}$ mice.

Comment 2.
The demonstration of BACE1 localization is not sufficient. Fig.5B is convincing, however, in Fig. 5D, it is very difficult to conclude that the colocalization of BACE1 and LAMP1 in Mgat3-deficient and wildtype brains is different. Other convincing methods are needed.

Response:
The other reviewer also raised this issue, and we have now analyzed BACE1 distribution using several other methods to strengthen our conclusion regarding altered BACE1 localization in the *Mgat3*$^{-/-}$ brain. First, consistent with the results obtained in *Mgat3*$^{-/-}$ MEFs, sucrose density fractionation experiments showed that more BACE1 is distributed to the lower density fraction in both 3-month-old (Fig. 5C) and 12-month-old *hAPP/Mgat3*$^{-/-}$ mouse brains (Expanded Fig. E4D) (page 9 lines 1-3 from the bottom), whereare the distribution of APP is unchanged. Because the same sucrose density fractionation method applied to both 3-month-old and 12-month-old mouse brains failed to demonstrate a difference in the former case, we used a more detailed fractionation method for 3-month-old mouse brains. Second, we double-stained APP and BACE1 in 12-month-old mouse brain sections, and found that co-localization of these two proteins was significantly decreased in *hAPP/Mgat3*$^{-/-}$ brains (Fig. 5D) (page 10 lines 1-2). Third, as suggested by reviewer 1, nicastrin was immunostained in brain sections and neurons as a control membrane protein. In contrast to BACE1 and lamp1, we confirmed that the co-localization of nicastrin with Lamp1 was not changed between *Mgat3*$^{+/+}$ and *Mgat3*$^{-/-}$ brains and neurons (Fig. 5G and Expanded Fig. E4F) (page 10 lines 8-12), supporting our conclusion that BACE1 localization is altered by *Mgat3* deficiency.

Taken together, these data support our conclusion that the reduction in Ab in *hAPP/Mgat3*$^{-/-}$ brain is caused by shift in localization of BACE1.

Comment 3
The results regarding Abeta generation, BACE1 activity and BACE1 degradation are not on the same line. Fig.1B shows BACE1 modification, Fig. 3 shows BACE1 activity and Fig.4 shows Abeta concentration in the brain. All these data is derived from 3-month-old APP mice. However, the authors described that there is no reduction of BACE1 proteins in 3-month-old APP-transgenic mice, which suggests that the altered Abeta generation is due to the different location of APP and BACE1. Thus, the title of this manuscript should be changed. The cortex and hippocampus should be used to investigate the location of APP and BACE1 instead of immortalized fibroblasts (Fig.5B).

Response:
We agreed that the altered BACE1 localization is more critical than the reduction in BACE1 protein level for the reduced Ab generation observed in *Mgat3*$^{-/-}$ mice. We have therefore changed the tile of the paper to “An aberrant sugar modification of BACE1 blocks its lysosomal targeting in Alzheimer’s disease”.

Furthermore, as mentioned above (response to comment 2), we confirmed that the localization of BACE1 is changed even in the brains of 3-month-old mice as shown by sucrose density fractionation experiments (Fig. 5C), and that co-localization of BACE1 and APP is decreased in the brain (Fig. 5D). These data support our conclusion that the lack of bisecting GlcNAc causes the reduction in co-localization of BACE1 with its substrate APP, leading to the decrease in Ab generation.

Immunostaining analysis of BACE1 with Lamp1 in the cortex and hippocampus has revealed that BACE1 signals at presynaptic terminals in the hippocampus are very potent (Kandalepas et al., Acta Neuropathol. 2013, 126, 329-352), which makes it difficult to quantify the co-localization rate of BACE1 and Lamp1. We therefore compared BACE1 protein levels in these regions between *hAPP/Mgat*3$^{+/+}$ and *hAPP/Mgat3*$^{-/-}$ mice. In the12-month-old *hAPP/Mgat3*$^{-/-}$ mice, we found a pronounced reduction in BACE1 in the cortex (Fig. R4). These results suggest that the

effect of bisecting GlcNAc on BACE1 localization in the brain is different in the cortex and hippocampus. This result is interesting but complicated, and the reasons for the regional specificity are unclear at present. Therefore, we would prefer to show the results of whole brains in Fig. 5C and Expanded Fig. E4D.

Fig. R4

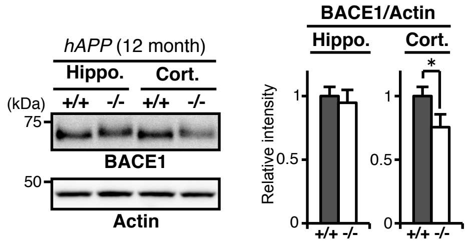

Hippocampus or cerebral cortex from *hAPP/Mgat3*$^{+/+}$ or *hAPP/Mgat3*$^{-/-}$ brains was homogenized, and proteins were blotted for BACE1. The BACE1 signal was then quantified. (*n* = 4)

Comment 4.
The protein level of BACE1 presented in Fig.5C could be equal between Mgat3-deficient and wildtype fibroblasts, which is controversial to Fig.6C. Because of no clear correlation between BACE1 modification and degradation, the reviewer wonders whether the hypothesis on BACE1 location in the late endosome is correct.

Response:
In the previous Fig. 5C (present Fig. 5E), immunoblot experiments from *Mgat3*$^{+/+}$ and *Mgat3*$^{-/-}$ MEFs were independently performed, and therefore direct comparison of BACE1 levels between *Mgat3*$^{+/+}$ and *Mgat3*$^{-/-}$ MEFs is not realistic. To avoid confusion, Fig. 5E has been replaced with data from another immunoblot to allow a direct comparison of BACE1 levels. The BACE1 levels are clearly reduced in *Mgat3*$^{-/-}$ MEFs in the new Fig. 5E. Moreover, quantification of the western blot results confirmed the reduction in BACE1 level as shown in Fig. 6C.

Comment 5.
The Abeta assay should be performed according to the standard protocols. Guanidine-soluble fraction does not reflect the membrane-associated Abeta. The results between the concentrations of Abeta in TBS-fraction and Guanidine-fraction might be larger. In Fig. 4C, no plaques in hAPP/Magt3-/- mice could be seen. It is very difficult to believe that the Abeta generation is nearly completely blocked when the sugar modification on BACE1 is ablated, whereas, in Fig. 5B, there are still sufficient BACE1 and APP located in the early endosome. It could be better for authors to present the location of APP and BACE1 in 12-month-old APP-transgenic mouse brain.

Response:
Our previous description of the Ab ELISA method might have been confusing, as we described "soluble" and "membrane" fractions in the method section. Because the "membrane" fraction actually includes both insoluble and membrane-associated Ab, we have rewritten our description of the method (page 19 lines 1-21). This method has been previously published (Saito et al., *Nat. Neurosci.*, 2014, 17, 661-663. Saito et al., *Nat. Neurosci.* 2011, 14, 1023-1032. Iwata et al., *J. Neurosci.*, 2004, 24, 991-998). We have also cited the relevant papers in the method section (page 19 line 2).

As mentioned above (response to comment 2), we have performed several experiments to determine the localization of BACE1 in *Mgat3*$^{-/-}$ brains. We carried out sucrose density fractionation experiments using both 3-month-old and 12-month-old mouse brains and found that the localization of BACE1 but not APP is altered even in the brains of 3-month-old mice (Fig. 5C), and that co-localization of BACE1 and APP is also decreased in 12-month-old mouse brains (Fig. 5D). These data support our conclusion that the lack of bisecting GlcNAc affects BACE1 distribution, leading to Ab reduction.

Comment 6.
The description of synaptic impairment and astrocytes is wrong in Fig. E3. Please use the published protocols.

Response:

We stained a synaptic marker or an astrocytic marker according to an already published protocol (Saito et al., *Nat. Neurosci.*, 2014, 17, 661-663), and we cited this paper in the results section on page 8 line 15. Furthermore, as suggested by reviewer 1, we have added new GFAP staining data which reveal a significant reduction in GFAP signals in *hAPP/Mgat3*$^{-/-}$ brains (Expanded Fig. E3C).

Comment 7.
The abbreviation, for example, E4-PHA on page 6, should be expanded when it was first used.

Response:
We are sorry for our poor explanation. We have defined the abbreviations where they first appear (e.g. GlcNAc, APP, ADAM, E4-PHA, PNGase, VPS35).

2nd Editorial Decision 21 November 2014

Thank you for the submission of your revised manuscript to EMBO Molecular Medicine. We have now received the enclosed reports from the Reviewers that were asked to re-assess it. As you will see while Reviewer 1 Reviewers is now globally supportive, Reviewer 2 is still somewhat reserved

Briefly, Reviewer 2 would prefer to see data from older mice due to a number of reasons s/he lists in his/her evaluation. The Reviewer is also asking to provide protein measurements for BACE1 and wonders whether there is BACE1 degradation in the lysosome. S/he would also like to see better and higher magnification images for Fig. E1.

Although I will not be asking you to provide further experimentation at this point, I would encourage you to 1) send me a rebuttal on the points raised; 2) provide the data if available, or in alternative amend your text as to avoid overreaching conclusions and 3) in any case provide improved images as indicated. I am willing to make an Editorial decision on your final, revised version, provided the issues raised are dealt with as mentioned above.

Please submit your revised manuscript within two weeks. I look forward to seeing a revised form of your manuscript as soon as possible.

***** Reviewer's comments *****

Referee #1 (Remarks):

The authors adequately addressed my previous concerns.

Referee #2 (Remarks):

Major comments:

1, The reviewer is still wondering the results from the cross-breeding between APP23 and Magt3 knockout mice. The manuscript presents the results from 3-month-old APP mice. However, APP23 mice show first rare deposits at 6 months of age (Sturchler-Pierrat et al., 1997). The demonstration of synaptic damage and gliaosis in Fig. E3 is obviously wrong. The authors should present a figure with at least one Abeta deposit in APP/Magt3-/- mice. The presentation in Fig. 4E is not sufficient, although there is publication showing cognitive deficits of APP23 mice in the water maze. The authors should also show whether deficiency of Magt3 affects cognitive functions of mice. Thus, it will be much better to show the data derived from 12-month-old APP mice instead of 3-month-old mice. At the current stage, the data presented in this manuscript is not sufficient to show effects of Magt3 deficiency on Abeta load, synaptic loss, neuroinflammation and cognitive function in the AD

mouse model;
2, The BACE1 activity should be presented dynamically according to the incubation time of tissue samples and BACE1 substrates. In stead of a 100min time point, the author should show the fluorescence intensity from 30min to 6h. Instead of purified BACE1, the membrane components should be prepared from cell lines and mouse brains (it will be much better to use cortex and hippocampus instead of the whole brain). Both BACE1 activity and protein levels should be measured. It could be necessary to use several antibodies from different origins to detect the protein amount of BACE1. The reviewer is clear that altered co-distribution between BACE1 and APP results in less Abeta production, whereas, the reviewer also sees the possibility of increased degradation of BACE1 in the lysosome;
3, When analyzing the co-distribution between APP and BACE1, the reviewer is more interested in the data derived from 12-month-old than 3-month-old APP mice, as the older APP mice develop AD-like pathology.

Minor comments:

In Fig. E1, C, a high magnification should be used and the clear morphology of microglia and oligodendrocytes should be demonstrated.

2nd Revision - authors' response 02 December 2014

***** Reviewer's comments *****

Referee #1 (Remarks):

The authors adequately addressed my previous concerns.

Response:

Thank you very much for reviewing our manuscript.

Referee #2 (Remarks):

Major comments:

Comment 1

The reviewer is still wondering the results from the cross-breeding between APP23 and Magt3 knockout mice. The manuscript presents the results from 3-month-old APP mice. However, APP23 mice show first rare deposits at 6 months of age (Sturchler-Pierrat et al., 1997). The demonstration of synaptic damage and gliaosis in Fig. E3 is obviously wrong. The authors should present a figure with at least one Abeta deposit in APP/Magt3-/- mice. The presentation in Fig. 4E is not sufficient, although there is publication showing cognitive deficits of APP23 mice in the water maze. The authors should also show whether deficiency of Magt3 affects cognitive functions of mice. Thus, it will be much better to show the data derived from 12-month-old APP mice instead of 3-month-old mice. At the current stage, the data presented in this manuscript is not sufficient to show effects of Magt3 deficiency on Abeta load, synaptic loss, neuroinflammation and cognitive function in the AD mouse model;

Response:

First, we would like to emphasize that old (12-month-old) mice and not 3-month-old mice were used for the experiments whose results are shown in Fig. E3 and Fig. 4E; this point is mentioned in the figure legends. As it seems that this misunderstanding has led to several criticisms, we have described "12-month-old" in Fig. 4E, 5D, 5F, 5G and E3. Our observation showing synaptic damage and gliosis in old mice (Fig. E3) is, therefore, reasonable (Sturchler-Pierrat et al., 1997). We found many reports in which the Y-maze is the only approach used to check cognitive deficit (Saito et al., Nature Neurosci. 14, 1023-32 (2011), Oakley et al., J. Neurosci. 26, 10129-40 (2006), Cho et al., Sci. Rep. 4:6777, (2014) in press), so we believe that we have adequately addressed this point. According to the reviewer's request, we have provided an additional image (Fig. R5) showing the Abeta load in APP/Mgat3-/- mice

Fig. R5

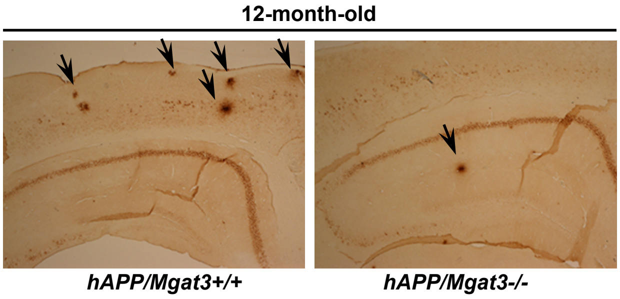

Comment 2

The BACE1 activity should be presented dynamically according to the incubation time of tissue samples and BACE1 substrates. In stead of a 100min time point, the author should show the fluorescence intensity from 30min to 6h. Instead of purified BACE1, the membrane components should be prepared from cell lines and mouse brains (it will be much better to use cortex and hippocampus instead of the whole brain). Both BACE1 activity and protein levels should be measured. It could be necessary to use several antibodies from different origins to detect the protein amount of BACE1. The reviewer is clear that altered co-distribution between BACE1 and APP results in less Abeta production, whereas, the reviewer also sees the possibility of increased degradation of BACE1 in the lysosome;

Response:

In order to assess the BACE1 activity, we performed several preliminary experiments in which recombinant BACE1 (1 – 600 ng) was incubated with the peptide substrate for various time periods. We have confirmed that for an incubation period up to 120 min, BACE1 hydrolyzes the substrate in a time- and dose-dependent manner. We have inserted a sentence to this effect in the "Materials and methods". From the immunoblot data in Fig. 5A, we estimated that the levels of immunoprecipitated BACE1 from the brains are less than 100 ng. The peptide substrate used in this study is known to be cleaved by other proteases, such as BACE2 and cathepsin D (Gruninger-Leitch *et al.* J Biol Chem 277, 4678 (2002)). Indeed, an in vitro BACE1 assay using a crude membrane extract resulted in a high background level, which makes it difficult to evaluate actual BACE1 activity (Fig. R6). Moreover, Fig. 7A shows that BACE1 substrates other than APP are normally cleaved in *Mgat3*$^{-/-}$ mice. Taken together, we can sufficiently conclude that the catalytic activity of BACE1 is properly maintained in *Mgat3*$^{-/-}$ mice.

Fig. R6

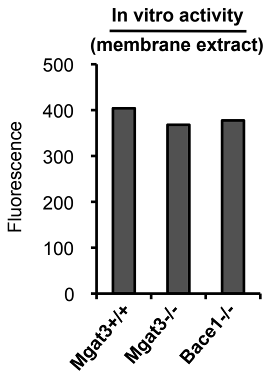

In preliminary experiments, we tried five kinds of anti-BACE1 antibodies for both immunoblot- and immunohistochemical analyses. Of these we found that only one anti-BACE1 antibody specifically detects BACE1 protein, as the signal clearly disappears in *Bace1*$^{-/-}$ mice (Fig. 5A). Furthermore, this antibody has also been validated by several groups using *Bace1*$^{-/-}$ mice (Rajapaksha et al., 2011, *Mol. Neurodegener*., Giusti-Rodriguez et al., 2011, *J. Neurosci*.).

As the reviewer mentioned, we have obtained data showing that the lysosomal degradation of BACE1 is regulated by bisecting GlcNAc. Even though we are planning to incorporate these data into another new manuscript, we have uploaded our data as a "related manuscript" [not shown] for reference. Briefly, we performed cycloheximide chase analyses to determine the BACE1 stability. When hydrogen peroxide was added to the culture media, BACE1 in Mgat3-/- cells was found to be more unstable compared with that in Mgat3+/+ cells. In contrast, the lysosomal inhibitor chloroquine effectively stabilized BACE1 in both Mgat3+/+ and Mgat3-/- cells. These data suggest that bisecting GlcNAc protects BACE1 from lysosomal degradation under the oxidative stress conditions.

Comment 3

When analyzing the co-distribution between APP and BACE1, the reviewer is more interested in the data derived from 12-month-old than 3-month-old APP mice, as the older APP mice develop AD-like pathology.

Response:

Again, "12-month-old" but not "3-month-old" mice were used for Fig. 5D experiments. We have mentioned the age of mice used in Fig. 5D to avoid confusion.

Minor comments:

In Fig. E1, C, a high magnification should be used and the clear morphology of microglia and oligodendrocytes should be demonstrated.

Response:

According to the reviewer's comment, we have added the magnified images in Fig. E1C.
